# Unmanned Aerial Vehicle Remote Sensing for Field-Based Crop Phenotyping: Current Status and Perspectives

**DOI:** 10.3389/fpls.2017.01111

**Published:** 2017-06-30

**Authors:** Guijun Yang, Jiangang Liu, Chunjiang Zhao, Zhenhong Li, Yanbo Huang, Haiyang Yu, Bo Xu, Xiaodong Yang, Dongmei Zhu, Xiaoyan Zhang, Ruyang Zhang, Haikuan Feng, Xiaoqing Zhao, Zhenhai Li, Heli Li, Hao Yang

**Affiliations:** ^1^Key Laboratory of Quantitative Remote Sensing in Agriculture of Ministry of Agriculture P. R. China, Beijing Research Center for Information Technology in AgricultureBeijing, China; ^2^National Engineering Research Center for Information Technology in AgricultureBeijing, China; ^3^Key Laboratory of Agri-informatics, Ministry of AgricultureBeijing, China; ^4^School of Civil Engineering and Geosciences, Newcastle UniversityNewcastle upon Tyne, United Kingdom; ^5^Crop Reduction Systems Research Unit, United States Department of Agriculture-Agricultural Research ServiceStoneville, NC, United States; ^6^Wheat Breeding Department, Institute of Agricultural Sciences for Lixiahe RegionJiangsu, China; ^7^National Center for Soybean Improvement, Nanjing Agricultural UniversityNanjing, China; ^8^Maize Research Center, Beijing Academy of Agriculture and Forestry SciencesBeijing, China

**Keywords:** UAV, remote sensing, high-throughput, field phenotyping, crop breeding

## Abstract

Phenotyping plays an important role in crop science research; the accurate and rapid acquisition of phenotypic information of plants or cells in different environments is helpful for exploring the inheritance and expression patterns of the genome to determine the association of genomic and phenotypic information to increase the crop yield. Traditional methods for acquiring crop traits, such as plant height, leaf color, leaf area index (LAI), chlorophyll content, biomass and yield, rely on manual sampling, which is time-consuming and laborious. Unmanned aerial vehicle remote sensing platforms (UAV-RSPs) equipped with different sensors have recently become an important approach for fast and non-destructive high throughput phenotyping and have the advantage of flexible and convenient operation, on-demand access to data and high spatial resolution. UAV-RSPs are a powerful tool for studying phenomics and genomics. As the methods and applications for field phenotyping using UAVs to users who willing to derive phenotypic parameters from large fields and tests with the minimum effort on field work and getting highly reliable results are necessary, the current status and perspectives on the topic of UAV-RSPs for field-based phenotyping were reviewed based on the literature survey of crop phenotyping using UAV-RSPs in the Web of Science™ Core Collection database and cases study by NERCITA. The reference for the selection of UAV platforms and remote sensing sensors, the commonly adopted methods and typical applications for analyzing phenotypic traits by UAV-RSPs, and the challenge for crop phenotyping by UAV-RSPs were considered. The review can provide theoretical and technical support to promote the applications of UAV-RSPs for crop phenotyping.

## Introduction

Crop production must double by 2050 to meet the predicted production demands of the global population (Ray et al., 2013). The crop yield demands are predicted to increase by 2.4% annually, but the average rate of increase is only 1.3%, with yields stagnating in up to 40% of land under cereal production (Fischer and Edmeades, 2010). To ensure improved agricultural productivity, to adapt to the impacts of climate change, and to prevent the resistance of pests and diseases to control measures, scientists must better understand the connection between a plant's observable characteristics (phenotype) and its genetic makeup (genotype). By establishing the connection between genotype and phenotype, it is possible to select high-yield stress-tolerant plants and improve agricultural production to satisfy the requirements of the growing human population (White et al., 2012; Li L. et al., 2014; Thorp et al., 2015). In the last two decades, gene sequencing of crops has proceeded at a rapid pace, but the translation of these data into the identification of desirable traits is constrained by the lack of knowledge of the associated phenotypes (Furbank and Tester, 2011; Zaman-Allah et al., 2015). To relieve this bottleneck and to fully benefit from the available genomic information, reliable, automatic, multifunctional, and high-throughput phenotyping platforms should be developed to offer plant scientists new insight into all the aspects of living plants. In recent years, rapid high-throughput phenotyping platforms (HTPPs) have been discussed (Yang et al., 2013; Araus and Cairns, 2014), and most are fully automated facilities in greenhouses or growth chambers with precise environmental control. Although HTPPs enable the capture of detailed, non-invasive information throughout the plant life cycle, the results from controlled environments are distinct from the actual situations that plants will experience in the field, making it difficult to extrapolate the data to the field.

Field-based phenotyping (FBP) is a critical component of crop improvement through genetics, as it is the ultimate expression of the relative effects of genetic factors, environmental factors, and their interaction on critical production traits, such as yield potential and tolerance to abiotic/biotic stresses (Araus and Cairns, 2014; Neilson et al., 2015). FBP is increasingly recognized as the only approach capable of delivering the required throughput and an accurate description of trait expression in real-world cropping systems. The performance of breeding programs on crop yield and productivity must be evaluated under natural conditions (Gonzalez-Recio et al., 2014; Gonzalez-Dugo et al., 2015; Rahaman et al., 2015). Currently, the most commonly field-based phenotyping platforms (FBPPs) use ground wheeled or aerial vehicles deploying multiple types of sensors to measure plant traits on a timescale of a few seconds per plot. For FBPPs based on ground vehicles, the process is time-consuming if there are too many plots needed to collect data (Zhang and Kovacs, 2012). For example, more than 40 h were required to cover the 20,000 plots with a single vehicle traveling at 2 km per h to measure traits on single rows (White et al., 2012). Using multiple vehicles and multiple sets of sensors to take measurements in all plots simultaneously would increase the costs (Zhang and Kovacs, 2012; Cobb et al., 2013). Moreover, FBPPs with ground vehicles cannot be used for cross-regional work due to the lack of maneuvrability. In the recent years, the cable-suspended field phenotyping platform was developed for rapid and non-destructive estimation of crop traits. For the cable-suspend field-based phenotyping platform, there's advantages of safety, high precision, independent of soil conditions and minimal tactile interference of plants. However, as it has to be located at certain sites, the coverable area of cable-suspend field phenotyping platform is relatively low, which limit its applications for the large-scale phenotyping (Kirchgessner et al., 2016). Some of these limitations can be addressed using satellite-based or aerial remote sensing approaches.

Satellite imaging technologies have become an extremely useful tool for collecting data for various agricultural applications (Li L. et al., 2014; Sankaran et al., 2015b). However, the major limitations of using the currently available satellite sensors are the high cost, the lack of spatial resolution for the identification of desirable traits, the risk of cloudy scenes and the long revisit periods (Issei et al., 2010; Gevaert et al., 2015). Alternatives based on manned airborne platforms have demonstrated capabilities for large-scale crop condition monitoring due to the high spatial and spectral resolutions of the sensors. However, in the case of breeding, and except for large seed companies, the high operating costs and the operational complexity have limited the use of these platforms to research activities (Chapman et al., 2014). Low-altitude and flexible UAV-RSPs are an important, affordable tool for crop phenotyping (Berni et al., 2009b; Liebisch et al., 2015)and precision agriculture (Hunt et al., 2005; Zhang and Kovacs, 2012; Ballesteros et al., 2014; Gomez-Candon et al., 2014; Candiago et al., 2015), and they provide a low-cost approach to meet the critical requirements of spatial, spectral, and temporal resolutions. In order to assess the precision and efficiency for field-based phenotyping in small plots by different remote sensing techniques, a direct comparison of three remote sensing approaches including UAV, proximal sensing, and satellite-based imagery was studied, which demonstrated that the UAV-based remote sensing performed best for acquiring canopy temperature and NDVI in breeding (Tattaris et al., 2016). Therefore, UAVs are becoming critical in crop phenotyping for high-throughput phenotyping of large numbers of plots and field trials in a near real-time and dynamic manner. UAVs can be used to execute autonomous tasks through the use of radio remote control equipment and an auto-control system, which can be divided into several types according to the flight mode (Sankaran et al., 2015b). Digital cameras, multispectral cameras, hyperspectral sensors, infrared thermal imagers, and light detection and ranging (LIDAR) are commonly deployed UAV-RSP sensors. The applications of these sensors for FBP include visible imaging for canopy surface modeling, crop height and biomass estimation (Mathews and Jensen, 2013; Diaz-Varela et al., 2014; Zarco-Tejada et al., 2014), visible–near-infrared spectroscopy to identify physiological status (Sugiura et al., 2005; Overgaard et al., 2010; Swain et al., 2010; Nigon et al., 2015), thermal imaging to detect water stress (Gonzalez-Dugo et al., 2013, 2014), LIDAR point cloud to measure plant fine-scale geometric parameters with high precision (Wallace et al., 2012), and microwave images for estimating soil moisture and canopy structure parameters by combining different spectral bands (Acevo-Herrera et al., 2010; Issei et al., 2010).

The crop phenotype is an expression of the genotype and the environment in which it grows, including geometric traits (e.g., plant height, LAI, lodging, crop canopy cover), canopy spectral texture (spectral features), physiological traits (e.g., chlorophyll, biomass, pigment content, photosynthesis), abiotic/biotic stress indicators (e.g., stomatal conductance, canopy temperature difference, leaf water potential, senescence index), nutrients (nitrogen concentration, protein content), and yield. Different methodological approaches based on spectra, canopy temperature, and visible light have been proposed to evaluate these traits in the field (Araus and Cairns, 2014). The geometric traits of a crop can be estimated by building the digital surface model (DSM) or digital elevation model (DEM) and conducting image classification analysis, which can be used to estimate the plant height, lodging area proportion, emergence, etc. (Hunt et al., 2005, 2010; Li J. W. et al., 2015). The absorption and reflectance characteristics of crops can be used to retrieve the physiological characteristics of a crop (Overgaard et al., 2010; Swain et al., 2010; Nigon et al., 2015). The canopy temperature is closely related to crop transpiration, which can reflect the leaf water potential, stomatal conductance, etc. under abiotic and biotic stress conditions. The combination of hyperspectral and thermal infrared data enables crop yield prediction (Berni et al., 2009b; Gonzalez-Dugo et al., 2015).

This review considers the latest technological aspects of remote sensing from the state-of-the-art of UAVs to estimate plant phenotyping parameters at the field-scale. The paper is organized as follows: (1) a literature survey of UAV remote sensing for field-based crop phenotyping in the last decade, (2) an overview of low-altitude UAVs and deployed sensors, (3) advances and applications of UAV remote sensing in field-based phenotyping, and (4) the limitations and future perspectives of UAV remote sensing for field-based crop phenotyping.

## Literature survey

**Figure 1:**
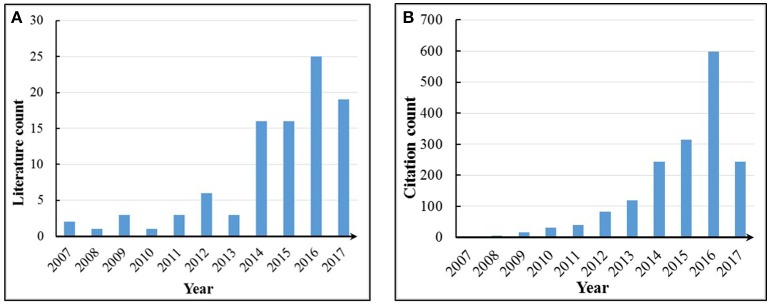
**(A)** Yearly literature count and **(B)** annual accumulated citation frequency for each article. The search was conducted on May 17, 2017.

There are 96 articles related to the keywords of “UAV,” “UAS,” “Drone,” “Unmanned Aerial Vehicle,” “Unmanned Aerial System,” “Unmanned Aircraft,” “Low Altitude Platform,” “Crop,” Plant,” “Crop breeding,” “Remote Sensing,” “Field-Based,” “Phenotyping,” and “Phenomics” in the Web of Science™ Core Collection Database (THOMSON REUTERS™) until May 17, 2017. However, there are only six articles that explicitly include “Phenotyping” or “Phenomics” in the titles and keyword (Zaman-Allah et al., 2015; Gomez-Candon et al., 2016; Haghighattalab et al., 2016; Holman et al., 2016; Shi et al., 2016; Watanabe et al., 2017). The other literatures are closely related to crop phenotyping using UAV-RSPs but do not explicitly mention crop phenotype; the research focuses on one or more crop traits. The number of published articles for each year is shown in Figure 1A. There's only one published articles during the period of 2005–2006. Most of the research focusing on field-based crop phenotyping has been performed since 2007 and has rapidly increased each year. A total of 85 articles were published during the period of 2012–2017, accounting for 88.5% of the total literature related to FBP using UAV-RSPs. The citations of retrieved articles during the period of 2007–2017 are given in Figure 1B, showing that the number of citations from 2012 to 2017 accounts for 94.4% of the total citations during that period. Considering the above literature statistics, field-based crop phenotyping has become a research hotspot.

**Figure 2:**
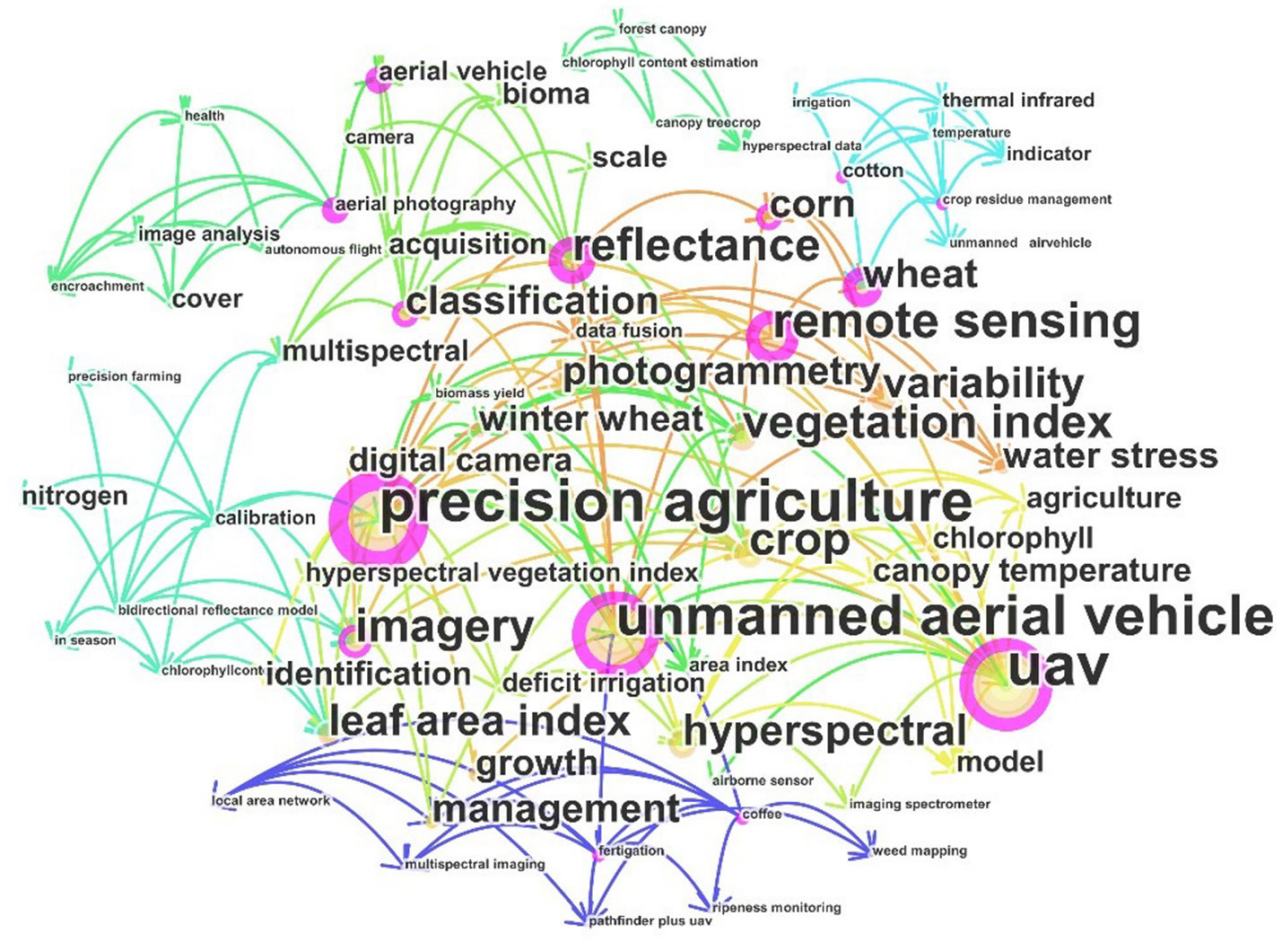
Frequency of keywords usage within total searched articles. The bigger font size, the more high frequency of usage, same as circle size corresponding to each keyword.The different color line just show connections among keywords or searched papers, respectively.

The journals that published at least three articles related to the topic of this review paper are shown in Table 1. The journal with the greatest amount of related research 〈〈Remote Sensing〉〉 published 17 articles, accounting for 17.7 % of all retrieved articles. The articles published in the journals of 〈〈Precision Agriculture〉〉 and 〈〈International Journal of Remote Sensing〉〉 are 8 and 7, respectively. Most of the related articles have been published in journals focused on remote sensing and agriculture, which is consistent with the fact that the agricultural model and remote sensing technology are the core science and technology for FBP by UAV-RSP. The retrieved articles were statistically analyzed using the analytical tool “CiteSpace” (Chen, 2004), and an analysis of the keyword frequency is shown in Figure 2. The most frequently used keywords include “Precision agriculture,” “unmanned aerial vehicle,” “UAV,” “Remote sensing,” and “vegetation index,” while “Phenotyping” and “Phenomics” were less frequently used. Even the research objectives of surveyed literatures focused on the crop growth monitoring, the crop phenotype includes numerous crop traits, such as traits related to the crop spectrum, structure, physiology, ecology, biotic stress, and abiotic stress (Pask et al., 2012). Thus, all the retrieved articles belonged to crop phenotyping by UAV-RSPs.

**Table 1:** Relevant journals that have published more than three papers related to UAV remote sensing for field-based crop phenotyping.

**Journals**	**Numbers of papers**
Computers and electronics in agriculture	3
IEEE Journal of selected topics in applied earth observations and remote sensing	3
International journal of agricultural and biological engineering	4
International journal of applied earth observation and geoinformation	3
International journal of remote sensing	7
Journal of applied remote sensing	5
Plos one	3
Precision agriculture	8
Remote sensing	17
Sensors	3

*The search was conducted on May 17, 2017*.

Based on the above analysis, the development of UAV-RSPs for crop phenotyping has gradually become a hot topic and can provide theoretical and technical support for precision agriculture and crop breeding. In addition, there are rare reports on FBP using UAV-RSPs in crop breeding (Issei et al., 2010; Torres-Sanchez et al., 2014, 2015). There is an urgent demand to develop strategies to rapidly and non-destructively acquire crop phenotypic data based on the current agricultural remote sensing technology. High-throughput field-based crop phenotyping can relieve the bottleneck of “linking genotype and phenotype” to accelerate the efficiency of crop breeding.

## UAV platforms and deployed sensors

### Overview of UAV-based phenotyping platforms

FBP using UAV-RSPs is based on an unmanned aircraft equipped with multiple sensors, using communication technology and GPS positioning technology to rapidly and non-destructively acquire high-resolution images about the crop canopy in the field. Remote sensing retrieval models are then used for phenotyping field trials after data processing (Sugiura et al., 2005; Li W. et al., 2016). The typical UAVs used for FBP include multi-rotors, helicopters, fixed-wing, blimps and flying wing (Table 2; Sankaran et al., 2015b) and are selected based on the purpose and budget. Blimps have the advantages of hovering ability, higher effective loads and the ability of vertical take-off and landing; however, they are slow because of the large size, and their stability is poor under windy conditions, making it difficult to obtain accurate information (Liebisch et al., 2015). Unmanned helicopters have the advantage of being able to take-off and land vertically, fly sideways, and hover. The helicopter payload is larger than that of a multi-rotor UAV and can support large sensors, such as LIDAR. However, the complex operation, lack of free hover, high maintenance cost and noise limit the application of helicopters (Sugiura et al., 2005; Swain et al., 2010; Chapman et al., 2014). The fixed-wing UAV is characterized by fast flying velocity and long flight time; however, the bottleneck for the fixed-wing application of FBP is the lack of free hover ability and the image blur caused by higher speeds and altitudes (Herwitz et al., 2004; Link et al., 2013). Multi-rotor UAVs have the advantages of low cost, the ability to hover, low take-off and landing requirements and are most frequently used for FBP. However, the greatest limitations of multi-rotor UAVs are the relatively short flight time, lower payload and the sensitivity to weather (Zhang and Kovacs, 2012; Pena et al., 2013; Uto et al., 2013; Nasi et al., 2015). Traditional UAV manufactured body materials are metals, such as aviation steel and aluminum (Colomina and Molina, 2014; Salami et al., 2014; Pajares, 2015). To reduce the UAV weight, enhance the body strength and prolong flight time, a variety of lightweight, high-strength composite materials have been widely used, including glass fiber and carbon fiber, and have become the main alternative materials for the body of UAVs. For the engine, the UAV engines can be divided into two categories: oil and electric engine. The oil engines have the advantages of strong wind resistance and long working time, while there're disadvantages of being bulky, producing big vibration and having poor reliability, which lead to the image blur (Xiang and Tian, 2011; Sankaran et al., 2015b). The electric engines have the advantages of safe, small vibration, easy to maintain and low cost, which make it become an important way for crop phenotyping by UAV; however, the short flight endurance time and weak wind resistance limited its use for crop phenotyping at large scale. A series of propulsion systems with the advantages of small volume, low vibration and new energy sources have become available and have greatly enhanced the UAV payload space and capacity. Hale engines and low-altitude silent propulsion systems are necessary to satisfy the requirements of small- and medium-size UAVs (Verhoeven, 2009).

**Table 2:** Typical types of UAVs used for field-based crop phenotyping.

**Specification**	**Description**				
	**Multi-rotor**	**Helicopter**	**Fixed-wing**	**Blimps**	**Flying wing**
	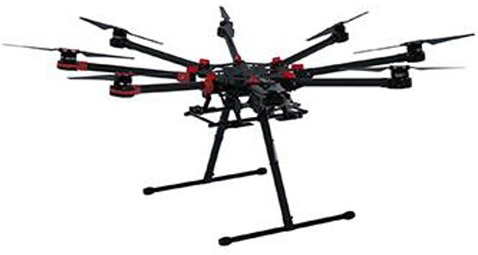	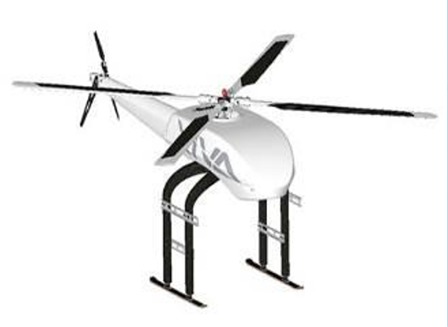	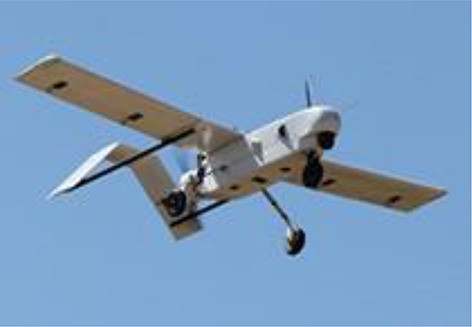	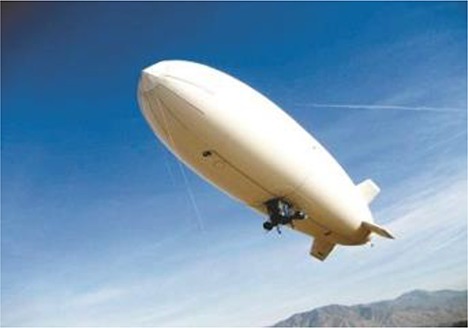	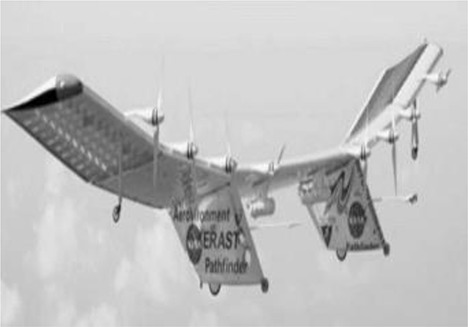
Model	DJIS1000	AXH-E230	Bat-3	CB3000	Pathfinder-Plus
Manufacturer	DJI technology	AVIX	MLB Co.	Beijing CSCA Co.	AeroVironment
Materials	Carbon fiber, High strength performance engineered plastics	Carbon fiber, aluminum alloy	Carbon fiber, engineered plastics	Kevlar fibers, fiber optic, electrical cores	Carbon fiber, Nomex, Kevlar, plastic sheeting, plastic foam
Cost	Low	Medium	Medium	High	very high
Power/Motors	Eight electric, 0.5 kw max each	One BLDC motors	Two-stroke engine	One oil engine	Eight (8) solar-electric, 1.5 kW max each
Gross weight/kg^a^	6	15	56	300	318
Payload capacity/kg^b^	7	15	9	10	67.5
Speed/m s^−1^	12	23	33	15	14
Endurance/h^c^	0.25	0.8	6	12	15
Altitude ceiling/m	500^d^	3,000	3,000	120	25,000

a Total weight with a battery;

b The payload including battery;

c Endurance with maximum payload;

d *The maximum flight height in China (the flight control system was restricted by the national regulations to set the flight height lower than 500 m)*.

**Figure 3:**
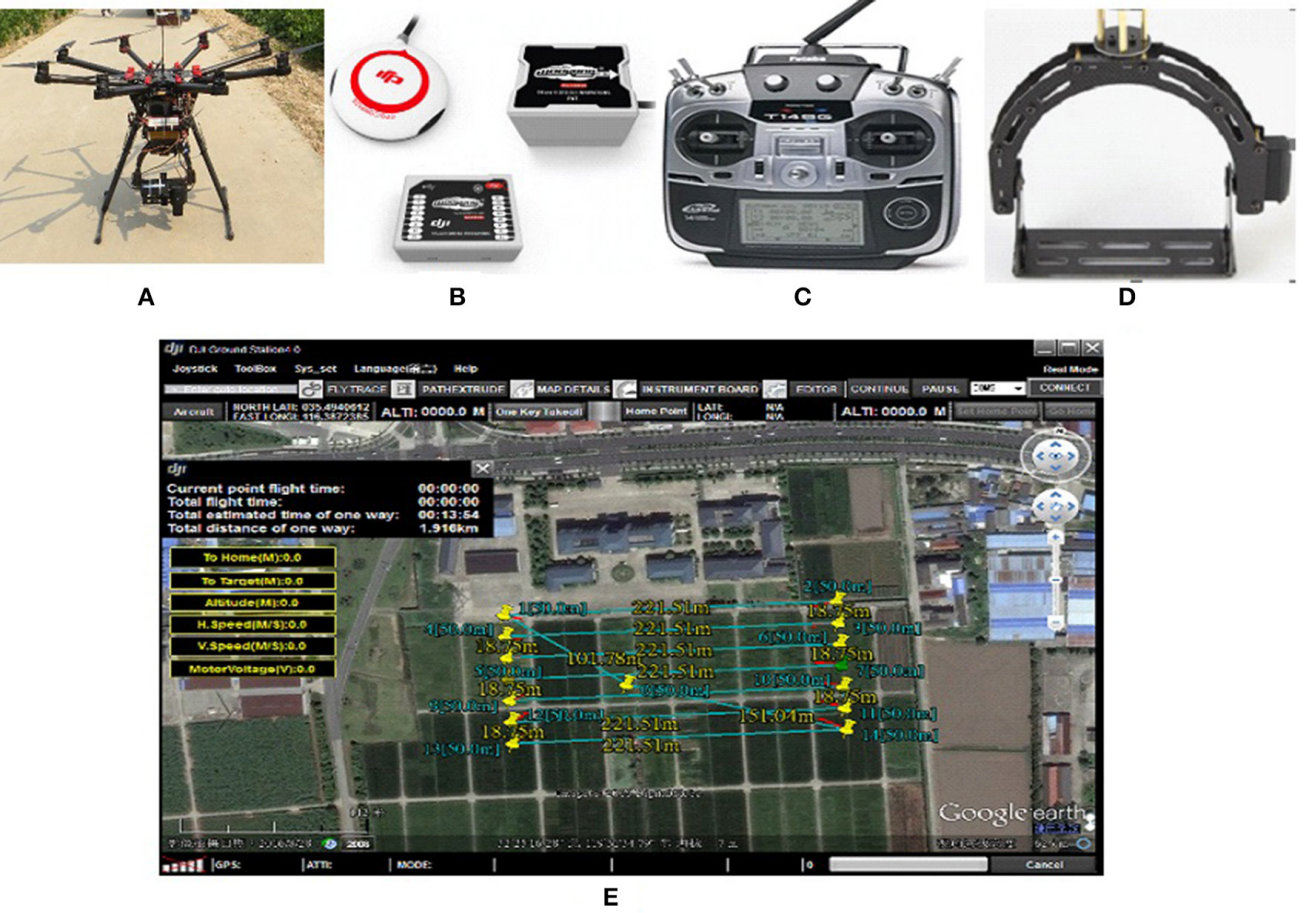
The components of a UAV adopted by NERCITA for field phenotyping in crop breeding. **(A)** An eight-rotors UAV named DJI Spreading Wings S1000+; **(B)** DJI flight control system named WOOKONG-M; **(C)** DJI Lightbridge 2 remote control; **(D)** Self-developed gimbal; **(E)** User interface of DJI Ground Station.

The UAV body, flight control system, remote control, sensors and oil/electric energy are the minimum required components for a UAV-RSP; while the ground station that enables the flight route planning and flight parameters setting, is an optional tool. The flight control system of a UAV is the core of the whole flight process, including take-off, flying in the air, executing tasks and recovery, and is one of the key technologies of a UAV system. Taking the UAV-RSP that was used for field phenotyping in crop breeding by NERCITA in China for example to illustrate the components of a UAV-RSP (Figure 3). The route planning tool of a UAV can set the flight height, flight speed, flight location and missions, and the flight details are transmitted to the flight control system through a data transceiver, which enables automatic take-off, the implementation of a default route, guided flight, and automatic landing.

### Sensors deployed by small UAVs

**Figure 4:**
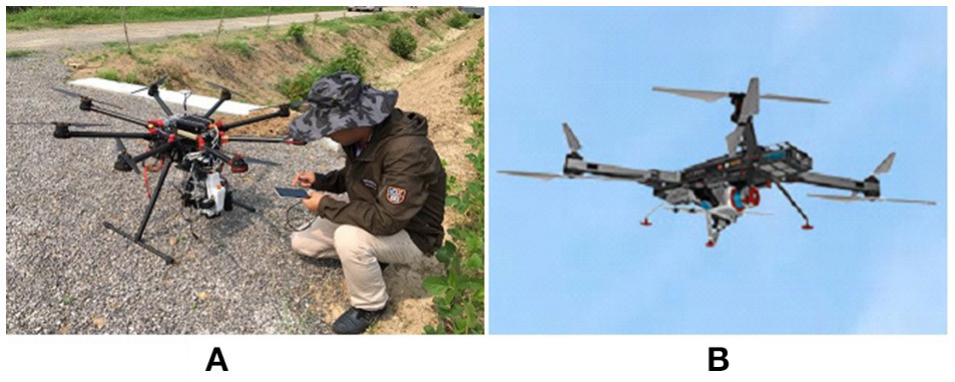
The UAV-RSPs adopted by NERCITA for field phenotyping in crop breeding. **(A)** A DJI Spreading Wings S1000+ equipped with hyperspectral imager (Cubert UHD185), thermal infrared imager (Optris PI400) and digital camera (Sony DSC-QX100); **(B)** A RIEGL RiCOPTER equipped with LIDAR (RIEGL VUX-1UAV) (Consent obtained from the individual for the publication).

UAV platform equipped with visible light imaging sensors, spectral sensors, infrared thermal sensors, fluorescence sensors, LIDAR et al. can obtain the color, texture, geometric contour of crops, which can be used to monitor plant height, LAI, biomass and other physiological traits of crops in different growth stages (Zhang and Kovacs, 2012; Rahaman et al., 2015; Table 3). As the equipped sensors are restricted by the UAV's payload capacity, which must meet the criteria of high precision, light weight, low power consumption and small size. Considering the cost, UAV payload and technological development of commercial products, digital camera (RGB), multispectral camera, infrared thermal imager, hyperspectral camera, LIDAR, three-dimensional camera and synthetic aperture radar (SAR) are the main sensors equipped by UAV-RSPs so far (Chapman et al., 2014; Sankaran et al., 2015b). The UAV-RSPs adopted by NERCITA were shown in Figure 4.

**Table 3:** Specifications and applications of typical UAV-deployed sensors.

**Type**	**Model**	**Weight (g)**	**Spectral bands**	**Wavelength range**	**Applications**	**Advantages**	**Disadvantages**	**References**
Digital camera	Sony DSC-QX100	179	Red, Green, Blue	–	Leaf color, plant height, lodging, canopy cover, fraction of intercepted radiation, LAI, 3D structure, leaf angle distribution	Low cost, light weight, convenient operation, simple data processing	Image amplitude, low radiometric resolution, lack of proper calibration	Samseemoung et al., 2012; Chapman et al., 2014; Torres-Sanchez et al., 2014; Guo et al., 2015
	Canon Ixus 110 IS RGB	145	Red, Green, Blue	–				
Multispectral camera	Tetracam ADC-Lite	200	Red, Green, NIR	520~920 nm	Leaf nitrogen content, yield, LAI, chlorophyll, biomass, weed, emergence, spring stand	Low cost, flexibility	Less bands, low spectral resolution and discontinuous spectrum	Overgaard et al., 2010; Swain et al., 2010; Nasi et al., 2015; Sankaran et al., 2015a; Vega et al., 2015
	Tetracam MCA-6	530	6	490~920 nm				
Hyperspectral imager	Cubert UHD185	470	125	450–950	Net photosynthesis, LAI, biomass, nitrogen, chlorophyll, yield, disease detection	More bands, high spectral resolution, ability of imaging	High cost, cumbersome data processing, sensitive to weather	Zarco-Tejada et al., 2013; Nigon et al., 2015
	HySpex VNIR-1600	4,600	160	400–1,000				
	Micro-Hyperspec VNIR model	700	324	380–1,000				
Thermal imager	FLIR Thermovision A40M	1,400	–	7.5–13 μm	Canopy temperature, stomatal conductance, water potential	Indirect determination of crop growth status under abiotic and biotic stress	Sensitive to the weather, frequent calibration, difficult to eliminate the influence of soil background	Berni et al., 2009b; Torres-Sanchez et al., 2015
LIDAR	RIEGL VUX-1UAV	3,500	NIR	–	Plant height, biomass	Rich point cloud information, Effective acquisition of high precision horizontal and vertical vegetation canopy structure parameters	High cost, Large data processing	Wallace et al., 2012; Wang et al., 2014

#### Digital camera

UAVs equipped with digital cameras can quickly acquire grayscale or color images to estimate crop height, leaf angel distribution, LAI, lodging and leaf color et al. (Ballesteros et al., 2014; Bendig et al., 2014; Chapman et al., 2014). RGB camera is the most commonly deployed by UAV in crop phenotyping research. The sensor has the advantages of low cost, light weight, convenient operation, simple data processing, and relatively low working environment requirements. Data can be collected under both sunny and cloudy conditions, but exposure should be set on the basis of the weather conditions to avoid inadequate or excessive image exposure. Unfortunately, this method is insufficient to accurately analyse crop phenotypic information for physiological traits due to the limitation of the less visible light bands.

The orthoimage or DSM/DEM is the ultimate product of aerial photography. For the digital images, as there's significant effect of interior orientation elements and camera distortion on image quality, the inspection and distortion processing of images according to the camera models are required. Then the homogeneous treatment for images is needed to enable the consistency of brightness, grayscale, and texture among images. The second step is to match the images according to the feature points of each image, the scale-invariant feature transform (SIFT) and random sample consensus (RANSAC) algorithm were adopted to process and optimize images. Finally, as the UAV can't acquire the images at large scale due to limitations of imaging devices and techniques, in order to evaluate the crop growth status throughout the study area, the orthoimage need to be generated using automatic splicing software, such as Photoscan, after aerial triangulation (Colomina and Molina, 2014). As each pixel value of images can be calculated from the reflectance or radiance of specific bands, then the color indices can be extracted with high-resolution images acquired by UAV to identify the vegetation features (Holman et al., 2016; Du and Noguchi, 2017).

#### Multispectral/hyperspectral imaging sensor

UAVs with spectral imaging sensors can obtain the spectral absorption and reflectance characteristics of crops, which can be used to monitor the crop planting area and crop growth, to evaluate the biological and physical characteristics of a crop, and to predict crop yield (Overgaard et al., 2010; Lebourgeois et al., 2012; Honkavaara et al., 2013; Candiago et al., 2015; Nigon et al., 2015). Multispectral and hyperspectral imaging sensors are the commonly deployed by UAV. Multispectral imaging sensors are defined as hardware that are capable of sensing and recording radiation from invisible as well as visible parts of the electromagnetic spectrum, which have been widely used for crop phenotyping due to the advantages of low cost, fast frame imaging and high work efficiency; however, they are limited by the low number of bands, low spectral resolution, and discontinuous spectrum (Berni et al., 2009b; Issei et al., 2010; Overgaard et al., 2010; Diaz-Varela et al., 2014; Candiago et al., 2015). Hyperspectral imaging sensors are cameras that can obtain a large number of very narrow bands and continuous spectra. Compared with multispectral imagers, hyperspectral imagers have the advantages of more band information and higher spectral resolution and can accurately reflect the spectral characteristics of the crop in the field and the spectral differences between crops (Zarco-Tejada et al., 2012, 2013; Colomina and Molina, 2014). In recent years, hyperspectral imaging has become a common way to acquire crop traits, such as crop water content, leaf nitrogen concentration, chlorophyll content, LAI, and other physical and chemical parameters, to facilitate crop yield prediction. Hyperspectral imaging technology is the future trend for crop phenotyping research using UAV-RSPs; however, the applicability of the physical inversion model based on hyperspectral remote sensing, the complex mechanisms of mixed spectral decomposition models for many kinds of field components (crop, soil, etc.) and an element extraction method require further research (Overgaard et al., 2010; Thorp et al., 2015). The pre-processing of spectral images mainly contains the radiometric calibration, geometric correction, image fusion and image enhancement. Then the spectral reflectance can be extracted using software, such as ArcGis, ENVI, etc. to build vegetation indices for retrieving crop phenotypic traits (Nasi et al., 2015).

#### Thermal infrared imaging sensor

Thermal infrared imaging sensors that using infrared detectors and an optical imaging lens to receive infrared radiation energy in the photosensitive element infrared detector can produce time series or single-time-point analysis based data, which have been widely used for crop growth monitoring and water stress detection (Gonzalez-Dugo et al., 2013, 2015). As the stomatal conductance, photosynthetic characteristics and transpiration rate are closely related to canopy temperature, the infrared thermal imaging technology can be used to determine the response of crops under stress conditions (Baluja et al., 2012). The conventional method for the determination of crop canopy temperature is using a handheld infrared thermometer, which is difficult to perform in the crop canopy temperature under different experimental conditions simultaneously, making it difficult to compare the difference in canopy temperature between treatments because the crop canopy temperature changes over time. In addition, the selection of the area for measurement is subjective and random (Berni et al., 2009b). UAVs equipped with infrared thermal imagers can quickly and non-destructively acquire the crop canopy temperature, which can effectively identify the temperature differences in the crop canopy under different environmental conditions. The thermal sensitivity is generally less than 80 mK. However, as the canopy temperature is sensitive to the environmental conditions, eliminating the influence of background temperature, including the incoming solar radiation, the ambient air temperature and the wind speed, is required (Sugiura et al., 2007; Deery et al., 2014). The most commonly adopted methods for achieving the goal include using sheet backgrounds to eliminate the background temperature, determining the percentage of bare soil and covered leaves in each image (Jones et al., 2009), and masking the data over a known background temperature (Chapman et al., 2014). As directly using the surface temperature of crop canopy for retrieving stomatal conductance or water potential is risky for the reason of influence soil pixels. In addition, canopy temperature on its own is not sufficient to derive stress, evapotranspiration or other similar parameters (Ortega-Farias et al., 2016). Therefore, it is needed to perform the whole energy balance, taking into account air temperature, wind speed, wind direction, etc. For the thermal infrared image processing, different methods including eliminating all pixels outside the expected temperature range for the leaves and using automated thresholding algorithms such as the Otsu method have been adopted to automatically extract canopy temperature (Gonzalez-Dugo et al., 2013). However, the presence of mixed pixels (pixel containing signals from both soil and vegetation) is a big problem for heterogeneous canopy (Baluja et al., 2012). The influence of mixed soil pixels can be reduced with thermal infrared data fusion with RGB image. However, the data fusion for image pre-processing is consuming and subjective.

#### LIDAR

LIDAR is a surveying method that measures distance to a target with emitting laser light. It is an active remote sensing device that uses the laser as the transmitting light source and adopts the photoelectric detection method. LIDAR is composed of a transmitter, receiver, tracking frame and information processing module, which has the advantages of high point density, high spatial resolution, smaller and lighter than traditional microwave radar, and good performance for low-altitude detection (Wallace et al., 2012). As the emitted pulse interacts with the canopy portions of it are returned by different elements, and the time delay between them provide the information about the horizontal and vertical canopy structure parameters. Simple LIDARs only measure first and last returns, while full-waveform ones return the photon density for a range of time delays. The limitations of LIDAR include the high cost, narrow beam, large data processing, and laser pulse can be totally absorbed by water, which greatly affect the popularity and application of LIDAR technology. LIDAR has been applied to estimate biomass and plant height of trees, and there are few applications of crop phenotyping using LIDAR (Ota et al., 2015). The methods used for extracting structural parameters in the forest are not suit for crop, because the plant height of crop is too low and the pattern of leaves aggregation was plant centered. Thus, it's necessary to explore strategies for extracting crop structural parameters by LIDAR.

#### SAR

SAR is an imaging radar used for conducting coherent processing of the received echo in different locations to obtain high-resolution data. SAR is a type of active microwave sensor that can be categorized into two types, focused and non-focused. SAR can obtain high-resolution radar images similar to optical images in very low visibility weather conditions and can work around the clock, which can be used for crop identification, crop acreage monitoring, key crop trait estimation and yield prediction, providing strong technical support for large-scale crop growth monitoring by remote sensing (Rosen et al., 2006; Wang et al., 2014).

In summary, UAVs deploy sensors with the advantages of flexible and convenient operation, on-demand access to data and high spatial resolution as an important means to rapidly and non-destructively acquire field-based crop phenotypes. However, because the remote sensing information from a single sensor is limited, combining multi-sensors to acquire and integrate data is necessary for field-based crop phenotyping by UAV-RSPs. In addition, as the image quality can be influence by wind speed, flight altitude and speed, sensor performance, aircraft vibration and image correction method etc., exploring strategies for acquiring images with high quality is necessary. The ability to efficiently process “big” remote sensing data acquired by UAV remains a challenge, as well as the ability to develop robust and fast algorithms according to the sensors used.

### Universal data processing methods

The pre-processing of remote sensing images is the basis for retrieving crop phenotype by UAV Remote Sensing. As there exist geometric and radiation distortion for remote sensing images, which were caused by the atmosphere, sensor characteristics, UAV attitude et al. Thus, it's necessary to eliminate the geometric and radiation distortion before retrieving crop phenotype by remote sensing images. Geometric and radiometric correction are two basic pre-processing techniques for UAV remote sensing data.

#### Geometric correction

There usually contains geometric deformation for the original images obtained by the UAV remote sensing platform due to the influence of the attitude and speed of UAV platform, the displacement of surface elevation model and the change of observed projection. Thus, the geometric correction of UAV remote sensing images is a prerequisite for subsequent data processing and analysis. The commonly adopted methods for geometric correction can be divided into two categories: (1) Geometric correction based on UAV POS Data (Yang et al., 2015); (2) Geometric correction based on high precision differential GPS (Saskia et al., 2017). The ground control points must be set for the traditional method, while the geometric correction can be achieved using low precision POS data of the UAV without setting ground points, which can improve the efficiency of UAV remote sensing images processing. DEM has been widely used in monitoring crop height and biomass, which usually can be generated with methods, such as moving surface fitting, multi-faceted function and finite element method for DEM interpolation (Liang et al., 2013).

#### Spectral radiation processing

The electromagnetic energy received by the UAV deployed sensors is inconsistent with the physical reflectance or the spectral radiation brightness of the target due to the atmospheric conditions, the physical characteristics of the sensor, the sun's position and the angle of measurement (Zhao et al., 2014). Therefore, in order to correctly reflect the spectral reflectance or radiation characteristics, radiation correction is required to eliminate or correct the various noise attached to the sensor's output radiant energy in the process of remote sensing imaging. Spectral radiation calibration refers to the process of converting a digital number (DN) of images acquired by UAV deployed sensors into physical parameters (Liang, 2008), such as radiation brightness, reflectance or surface temperature, which includes relative calibration and absolute calibration. The commonly adopted reflectance conversion methods include linear regression model, flat field model, internal mean method, logarithmic residual model and dark target method et al. The atmospheric correction can be neglected as the atmosphere between the surface and the sensor has a weak influence on the radiation of the sensor entrance with the relatively low flight attitude of micro-UAV (Hernandez-Lopez et al., 2012). The spectral reflectance or vegetation indices derived from hyperspectral images were influenced by the angular view of UAV-deployed sensors. Radiative transfer models can be act as useful tools for correcting angular influences over vegetated environments (Burkart et al., 2015). Spectral feature extraction is the process of the decomposition, reconstruction and selection of the spectral measurement, which can be divided into three categories, namely the statistical reduction method, the characteristic spectral line method and the spectral line method according to the characteristic expression formula (Li, 2012). The statistical reduction method is the most widely used method for spectral feature extraction, which has the advantages of easy to operate and use, while there's the disadvantage of lack of physical meaning. The commonly used statistical reduction methods include principal component analysis, wavelet transform, manifold learning and supervised correlation vector machine, support vector machine and discriminant analysis (Laliberte et al., 2011). The essence of the method is the decomposition, reorganization and selection of the celestial radiant energy in order to remove redundant noise, and convert the signal into the pattern easy to process in the subsequent application. The advantage of the feature spectrum method is that the good physical meaning, but it is often computationally intensive and inefficient. The results lack of representation for the spectral characteristics when there's more spectral times and less observation data. There's also the advantage of strong physical meaning for the spectral line method, however, it's sensitive to the spectral complexity and instrument calibration (Liang et al., 2013).

#### Universal modeling methods

The main adopted methods for crop phenotyping by UAV remote sensing include image analysis, physical model method, empirical statistic method and advanced data analysis method, such as machine learning (Liang et al., 2013; Bendig et al., 2015). As the modern imaging techniques can allow for the visualization of multi-dimensional and multi-parameter data, the high-resolution images acquired by UAV remote sensing platform have been used for estimating crop structure characteristics using image analysis method (Torres-Sanchez et al., 2015). The objected-based image analysis method works with groups of homogeneous and contiguous pixels, which can help to solve the problem that the spectral similarity of crop and weed pixels in early growth stages (Sankaran et al., 2015b). The general description of the procedure for image analysis include image acquisition, segmentation and classification (Mathews, 2014). Imaging analysis algorithms are the primary drivers for extracting statistical data to quantify the phenotype. Typical segmentation algorithms are based on a color model and threshold value (Li L. et al., 2014). In order to accelerate the working efficiency, the automatic object-based methods have been highlighted as the UAV can acquired massive image data. The thresholding OBIA algorithm is essential for the automatic vegetation classification (Torres-Sanchez et al., 2015). As the physical inversion model, such as radiative transfer model, refer to complex problems of the leaf and canopy structure, radiation transmission, the combination of radiative transfer mechanism and spectral absorption characteristics of biochemical component is required to retrieve crop phenotype. For example, the PROSAIL model combined with spectral data were used for monitoring the LAI of wheat (Bendig et al., 2015). The crop phenotypic traits, such as crop canopy cover, LAI, chlorophyll content, plant nutrient, water content, biomass and yield et al., can be rapidly acquired using empirical statistical models with various vegetation indices. The commonly adopted empirical statistical methods for high-throughput field phenotyping include multiple linear regression, partial least squares regression and stepwise linear regression (Richards, 1990). Using advanced data analysis methods, such as principal component analysis (PCA), artificial neural network (ANN), support vector machine (SVM), and wavelet analysis (WA) et al., can be act as an important method to improve the prediction accuracy of the retrieval models. However, there're disadvantages of the lack of explicit regression relations, the time-consuming calculation process, which greatly limit its efficiency and application scope (Zhao et al., 2014). The empirical statistical models are widely used and effective in the research of UAV Remote Sensing for field-based phenotyping according to the literature survey, but the applications were limited by the higher demand on practical surveying data and lack of physical meaning. For the machine learning, the obvious drawback is the lack of the ability to interpret data, which make it difficult to exploit the advantages of machine learning.

## Advances of UAV remote sensing in field phenotyping

The crop phenotype is defined as the physiological and biochemical characteristics and traits that are influenced by the genetic information of the crop and environmental factors, and it can be divided into different levels, such as groups, individuals, organs, tissues and cells (Cobb et al., 2013; Yang et al., 2013; Araus and Cairns, 2014). The crop phenotype is closely related to crop production and crop breeding. Rapid and non-destructive acquisition of the phenotypic information of crops in the field is an important prerequisite for studying the genetic inheritance law and accelerating the efficiency of large-scale crop breeding (Yang et al., 2013). The traditional methods for measuring crop traits, such as biomass, LAI and yield, depend on manual sampling, which is time consuming, inefficient, and inaccurate (Berni et al., 2009b; Rahaman et al., 2015; Li W. et al., 2016). There are definite advantages for field-based crop phenotyping based on UAV-RSPs, including high technical efficiency, low cost, suitability for complex field environments, timely field work, high-resolution data acquisition, rapid identification of growth information, synchronous image acquisition, and high operating efficiency. UAV-RSPs have been widely used for field-based crop phenotyping as an important tool for high-throughput phenotyping to assist crop research (Table 4). However, there's still the lack of validation of field-based phenotyping by UAV remote sensing with massive germplasm (over 1,000 plots).

**Table 4:** Typical applications of field-based crop phenotyping by UAV-RSPs.

**UAV platform**	**Sensors**		**Crop**	**Flight altitude**	**Research objects**	**Methods**	**Indices**	**Variables**	**Best performance**	**References**
	**Type**	**Model**								
Airborne	LiDAR	Leica ALS70	Maize	1,500 m	Plant height estimation	Image analysis	Normalized point heights	Plant height	*R*^2^ = 0.79	Li Z. et al., 2014; Li W. et al., 2015
					LAI estimation	Beer–Lambert equation	Laser intensities	LAI	*R*^2^ = 0.78	
					Biomass estimation	Structural equation modeling	Crop height, LAI	Aboveground biomass	*R*^2^ = 0.82	
	Hyperspectral camera	AISA-Eagle	Potato	1,900 m	Leaf N concentration detection	Quantitative inversion model	Nitrogen Sufficiency Index (NSI)	Leaf N concentration	*R*^2^^*^ = 0.79	Nigon et al., 2015
Fixed-wing UAV	Digital Camera	Ricoh GXR A12	Maize	375 m	Lodging estimation	Image analysis	RGB gray level, optimum features	Lodging area	about 99.7 %	Li Z. et al., 2014
	Hyperspectral camera	Micro-Hyperspec VNIR	Vineyards	575 m	Estimation of net photosynthesis	Fraunhofer Line Depth (FLD) principle based on three spectral bands	FLD3 [Three bands for the in (L763 nm) and out bands (L750 nm; L780 nm)]	Net photosynthesis	*R*^2^ = 0.52	Colomina and Molina, 2014
	Multispectral Camera	Tetracam ADC-Lite	Maize	150 m	Low-nitrogen stress detection and grain yield prediction	Quantitative inversion model	Normalized Difference Vegetation Index (NDVI), Canopy Structure Index (CSI)	Yield	*R*^2^ = 0.40	Overgaard et al., 2010
	Multispectral Camera	Canon S110 NIR	Weed	115 m	Weed detection	Image Analysis	Three UAV bands and texture layer	*Silybum marianum* (L.)	overall accuracy of 87.04%	Tamouridou et al., 2017
	Multispectral Camera	Tetracam MCA-6	Peach	150 m	Mapping radiatio n interception	Image Analysis, radiative transfermodel inversion	NDVI	fIPAR	*R*^2^ = 0.85	Guillen-Climent et al., 2012
			citrus						*R*^2^ = 0.84	
	Hyperspectral camera	Micro-Hyperspec VNI	Citrus	575 m	Water stress detection	Quantitative inversion model	Photochemical Reflectance Index (PRI)	Stomatal conductance	*R*^2^ = 0.59	Zarco-Tejada et al., 2012
	Thermal Camera	Miricle 307					Crown temperature		*R*^2^ = 0.78	
Flying wing	Multispectral Camera	DuncanTech MS3100	Cherries	6,400 m	Agricultural surveillance and decision support	Quantitative inversion model	Pixels with channel 3 (ch3)/Pixels with channel 2 (ch2)	Mature ratio	*R*^2^ = 0.81	Herwitz et al., 2004
Helicopter	Digital Camera	Ricoh GR Digital III/IV	Sorghum	60 m	Ground cover estimation	Image Analysis	–	Ground cover	*R*^2^ = 0.77	Chapman et al., 2014
	Multispectral Camera	Tetracam ADC	Rice	20 m	Yield prediction	Quantitative inversion model	NDVI	Yield	*R*^2^ = 0.72	Swain et al., 2010
					Total biomass estimation			Biomass	*R*^2^ = 0.75	
	Multispectral Camera	Tetracam	Corn	150–200 m	LAI and Chlorop hyll estimation	Quantitative inversion model	NDVI	LAI	*R*^2^ = 0.50	Berni et al., 2009b
		MCA-6	Olive, Peach				TCARI/OSAVI	Chlorophyll concentration	*R*^2^ = 0.88	
	Thermal Camera	FLIR Thermovision A40M	Olive	1,000 m	Mapping canopy conductance	Energy balance model	Canopy temperature	Canopy conductance	*R*^2^ = 0.61	Berni et al., 2009a
Multi-rotor UAV	Digital Camera	Digital photography camera PENTAX A40	Onion	40 m	LAI estimation	Image Analysis	Canopy visual scores	LAI	*R*^2^ = 0.83	Corcoles et al., 2013
	Digital Camera	Panasonic Lumix GX1	Barley	50 m	Plant height and biomass estimation	Image Analysis	Crop surface model	Plant height	*R*^2^ = 0.92	Bendig et al., 2014
								Fresh biomass	*R*^2^ = 0.81	
								Dry biomass	*R*^2^ = 0.82	
	Digital Camera	Aeryon Photo3S	Soybean	120 m	Crop growth monitoring	Image analysis	–	Lodgin	–	Zhang et al., 2014
	Multispectral Camera	Tetracam ADC-Lite					NDVI	Fall armyworm		
	Digital Camera/Multispectral Camrea	Pentax Optio A40; Tetracam ADC	Maize Onion	25 m	Green canopy cover and LAI estimation	Quantitative inversion model	VARI_green_	Green canopy cover	*R*^2^ = 0.94 *R*^2^ = 0.96	Ballesteros et al., 2014
	Digital Camera/Multispectral Camrea	Olympus PEN E-PM1; Tetracam mini-MCA-6	Maize, Sunflower and Wheat	30 m	Vegetation detection	Object Based Image Analysis	ExG and NDVI	Crop classification errors	between 0% and 10%	Torres-Sanchez et al., 2015
	Digital Camera	Sony NEX 7	Wheat	45 m	Growth monitoring	Image Analysis	Crop surface model	Crop height	*R*^2^ = 0.99	Holman et al., 2016
	Digital Camera	SONY ILCE-6000	Wheat	100 m	Growth monitoring	Image Analysis	VDVI, NGBDI, GRRI, ExG	Yield	*R*^2^ = 0.94	Du and Noguchi, 2017
	Hyperspectral camera	Developed 256-band Hyperspectral Sensor	Rice	10 m	Chlorophyll Density estimation	Quantitative inversion model	Red-edge (RE) and near-infrared (NIR) spectral	Chlorophyll density	*R*^2^ = 0.64	Uto et al., 2013
	Hyperspectral camera	Cubert UHD185	Barley	30 m	Vegetation monitoring	Quantitative inversion model	BGI2	Chlorophyll	*R*^2^ = 0.52	Aasen et al., 2015
							RDVI	LAI	*R*^2^ = 0.32	
							RDVI	Fresh biomass	*R*^2^ = 0.29	
	Hyperspectral Camera/Thermal Camera	Micro-Hyperspec VNIR,	Wheat	345 m	Physiological Conditions assessment	Quantitative inversion model	Modified soil-adjusted indices (MSAVI)	Yield	*R*^2^ = 0.31	Gonzalez-Dugo et al., 2015
		FLIR SC655				Quantitative inversion model	Crop Water Stress Index (CWSI)	Yield	*R*^2^ = 0.53	
						Information fusion of Multi-sources remote sensing	CWSI, FLD, PRI	Yield	*R*^2^ = 0.77	
	Multispectral Camera	Tetracam ADC-Lite	Sunflower	75 m	Phenotypic analysis	Quantitative inversion model	NDVI	Yield	*R*^2^ = 0.74	Rosen et al., 2006
								Biomass	*R*^2^ = 0.90	
								Nitrogen content	*R*^2^ = 0.96	
	Multispectral Camera	XNiteCanon SX230 NDVI	Wheat	100 m	Crop growth monitoring	Image analysis	GNDVI	Emergence	*R*^2^ = 0.76	Zhang et al., 2014
								Spring stand	*R*^2^ = 0.74	
	Multispectral Camera	Tetracam miniMCA6	Citrus	100 m	Huanglongbing (HLB) detection	Quantitative inversion model	NDVI, GNDVI, SAVI, NIR, R	Classification accuracy	about 85%	Garcia-Ruiz et al., 2013
	Multispectral Camera	Tetracam ADC-lite	Vineyard	150 m	Vineyard detection: mapping crop variability indices	Image Analysis, quantitative inversion model	NDVI	Vineyard variability indices	higher than 95%,	Comba et al., 2017

* *Coefficient of determination (R^2^)*.

### Crop geometric traits

**Figure 5:**
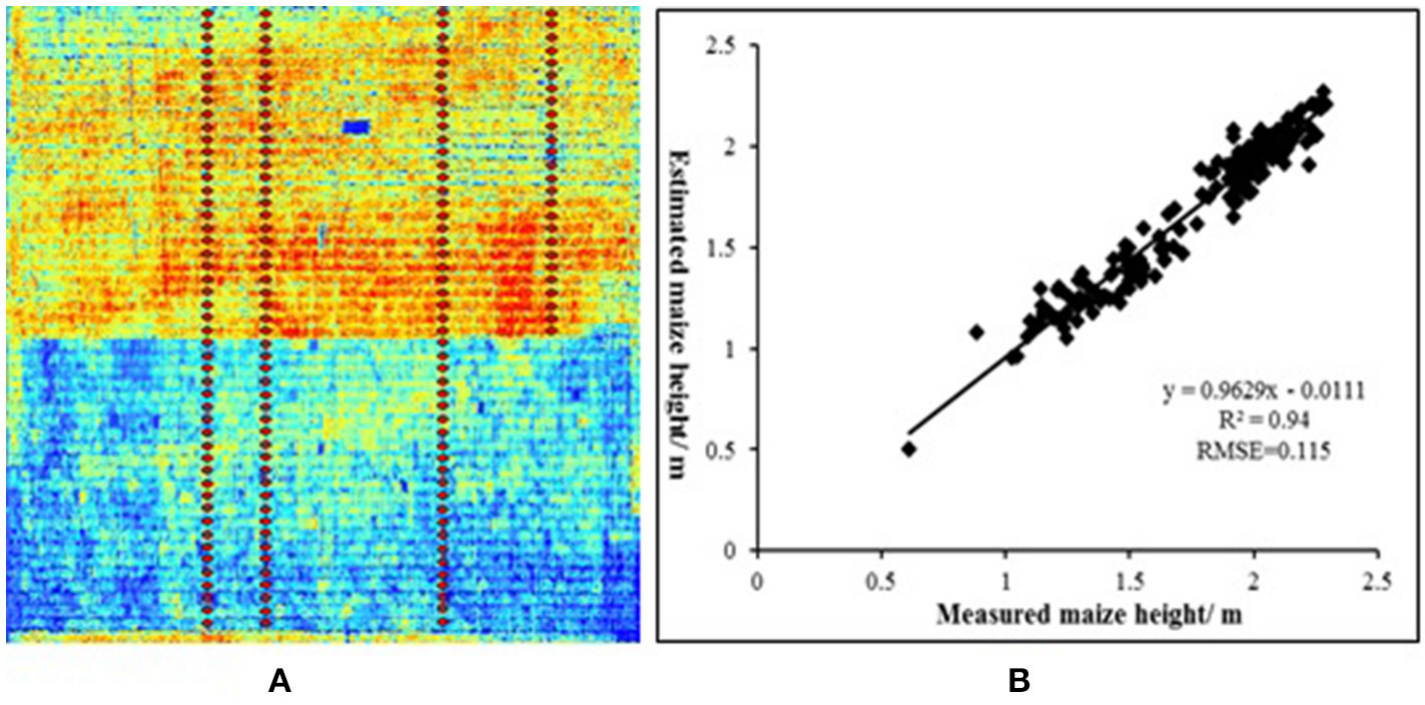
The estimation of plant height of summer maize. **(A)** Crop height model (CHM) from LIDAR in July 8, 2016 (red points indicates the measured sample points), **(B)** Validation of maize height from LIDAR.

Geometric traits, such as crop height (Bareth et al., 2016; Holman et al., 2016), vegetation cover fraction (Weiss and Baret, 2017; Yu et al., 2017), fraction of intercepted radiation (Guillen-Climent et al., 2014; Duan et al., 2017), LAI (Corcoles et al., 2013), lodging (Chapman et al., 2014), 3D structure (Aasen et al., 2015; Weiss and Baret, 2017), leaf angle distribution (Guo et al., 2015; McNeil et al., 2016), tiller densities (Du and Noguchi, 2017), and emergence (Sankaran et al., 2015a), can be rapidly obtained using the image analysis methods or the spectral and texture information in the images acquired by a UAV deployed imaging sensors (Tamouridou et al., 2017; Yu et al., 2017). The densified three-dimensional point clouds can be created using structure from motion (SFM) based on the images acquired by a UAV equipped with a digital camera (Turner et al., 2012; Holman et al., 2016). Then, the DSM and DEM are extracted to generate crop surface models (CSMs) (De Souza et al., 2017), which can be used for lodging area estimation (Chapman et al., 2014) and plant height monitoring (Li W. et al., 2016). The accuracy of plant height estimation using DSM and DEM can be significant improved with the use of Real Time Kinematic (RTK) GPS (Xiong et al., 2017). The plant height of maize was estimated by UAV equipped a digital camera with the R^2^ and NRMSE of 0.88 and 6.40%, respectively (Li W. et al., 2016). In addition to estimating plant height using DEM generated by digital images, the point clouds acquired by LIDAR can also be used for estimating plant height. In NERCITA's ongoing study, 69 (Inbred) and 104 (Hybrid) maize lines were selected for estimating plant height with UAV-based LIDAR. The determination coefficients between the estimated maize height by LIDAR and measured plant height can reach to 0.94 in maize breeding (Figure 5), which showed a high accuracy for plant height estimation in breeding. Flowering dynamics act as an important phenotypic trait for paddy rice, which is time-consuming and labor-intensive by manual observation. The image analysis technique including scale-invariant feature transform descriptors and machine learning performed well for detecting flowering panicles with RGB images (Guo et al., 2015). However, the applications of UAV for counting flowering panicles haven't been reported. Automated characterization of flowering dynamics by UAV remote sensing at large-scale is essential for accelerating the breeding process. Thus, there's still potential for wider applications of field-based phenotyping by UAV with the advance of image analysis method, low-cost sensor and effective image processing software.

Classification of remote sensing images is an important part of image feature analysis and can be used for leaf color monitoring and crop identification (Geipel et al., 2014). Classification methods can be divided into supervised and non-supervised classification according to the level of user intervention required. Supervised classification methods include maximum likelihood discriminant, neural network classification, fuzzy classification, minimum distance classification and Fisher classification, while unsupervised classification methods include dynamic clustering, fuzzy clustering, hierarchical clustering and splitting (Zhao and Qian, 2004). Ground canopy cover (GCC) was recognized as an important parameter related to the crop photosynthesis and transpiration (Mullan and Reynolds, 2010). GCC is dynamic during the crop growth stages. GCC reduced as a result of leaf rolling or wilting under drought stress conditions (Sayed et al., 2012), which can be used for studying the response of crop varieties under abiotic/biotic stress. Thus, the dynamics of GCC over time have been regarded as one of the targeted phenotypic traits in crop breeding (Zaman-Allah et al., 2015; Yu et al., 2017). Ground canopy cover can be estimated by canopy reflectance, surface temperature and imagery (Booth et al., 2005; Rajan and Maas, 2009). For example, Champan acquired the GCC of sorghum with best linear unbiased prediction using a UAV with a digital camera and showed that the UAV-RSP has great potential for crop phenotyping in specific breeding plots (Chapman et al., 2014). As the accuracy of GCC estimation is relatively low using NDVI at early growth stage due to the influence of soil. The pixel-level data extracted from images with high resolution acquired by UAV performed better for GCC estimation (Sankaran et al., 2015b). The large amount of data acquired by UAV-RSPs deploying multi-source sensors can be rapidly and efficiently processed through machine learning, which has been widely used in the field of crop phenotyping under stress conditions. Identification, classification, quantification and prediction are the main steps for analyzing the physiological traits under conditions of biotic and abiotic stress based on machine learning (Zhao and Qian, 2004; Singh et al., 2016).

LAI reflects the growth status of the crop population, and it is closely related to crop yield. Methods for estimating LAI by remote sensing include statistical models and optical models. Vegetation indices can be built after processing the spectral reflectance data acquired by UAV-based spectrometers using statistical methods to estimate LAI, such as Normalized Difference Vegetation Index (NDVI), Ratio Vegetation Index (RVI) and Perpendicular Vegetation Index (PVI). For example, the LAI in soybean breeding was estimated based on UAV-based hyperspectral by NERCITA, with the determination of coefficients and RMSE for calibration model of 0.70 and 0.67, respectively, which showed a good precision (Lu et al., 2016). In addition to estimate LAI with statistical models based on vegetation indices, the radiation transmission model can also be used for LAI estimation. For example, the PROSAIL model combined with spectral data in the field was used to estimate the LAI of wheat (Vega et al., 2015).

### Crop phenotyping related vegetation spectral indices

**Figure 6:**
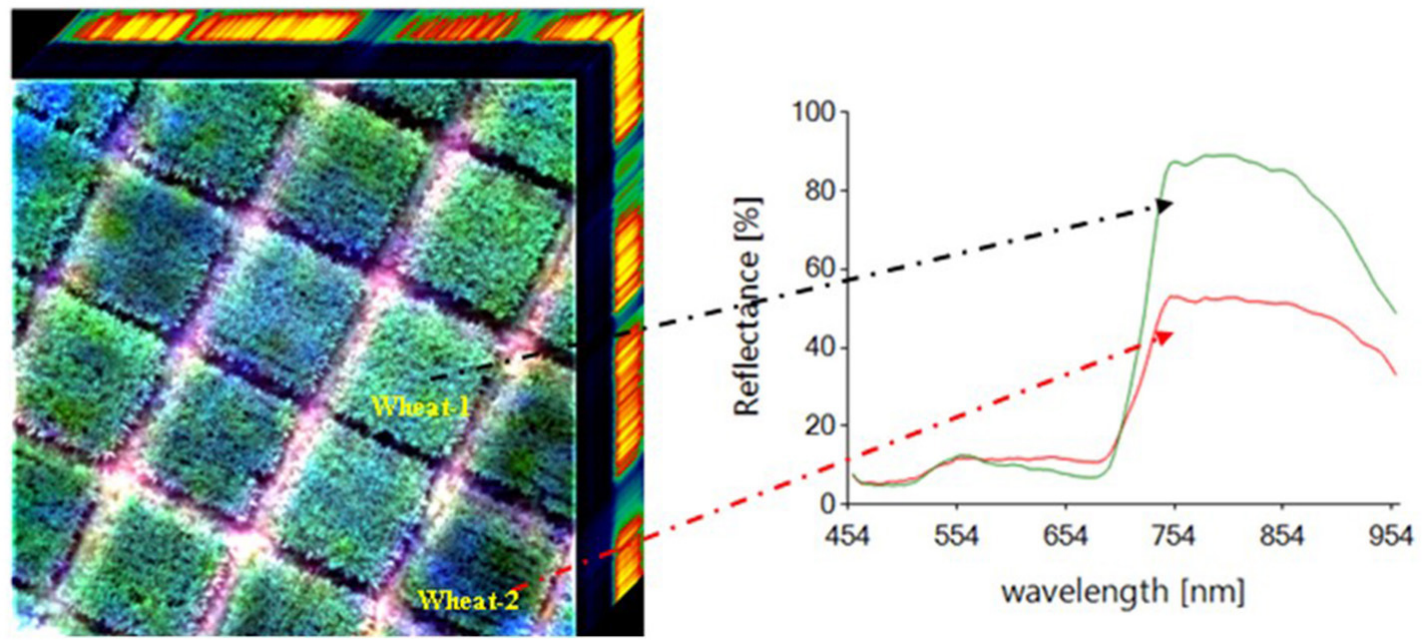
The distribution of hyperspectral imaging in wheat breeding.

**Figure 7:**
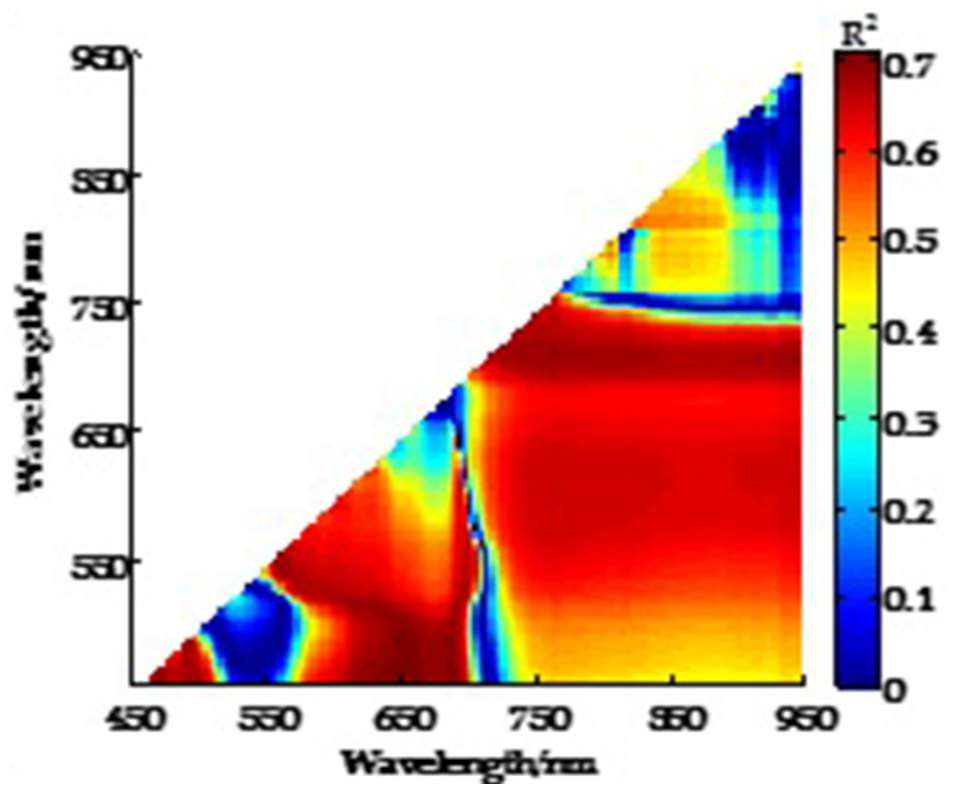
Coefficient of determination (*R*^2^) between LAI and NDSI calculated from all possible two-band combinations at jointing and flowering stages of wheat.

The absorption and reflection characteristics differ between spectral bands in the crop leaves, with strong absorption in the visible band and strong reflection in the near-infrared band, providing the physical basis of crop growth monitoring by remote sensing. The reflection characteristics of crop leaves in different bands can be acquired by the imaging spectrometer (Figure 6). A large number of vegetation indices can be constructed by the empirical treatment of spectral reflectance data at different wavelengths, which can reflect the difference between the reflectance of visible light, near-infrared and soil background, indicating the crop growth status (Table 5). For example, the relationship between LAI and the normalized difference spectral index (NDSI) calculated from all possible two-band combinations was evaluated and showed that the NDSI consists of two sensitive bands and can be used to estimate LAI with high accuracy (Figure 7; Gao et al., 2016). The spectral characteristics of the crop canopy can be rapidly acquired by a UAV-RSP equipped with multispectral and hyperspectral sensors and can then be processed to build a vegetation index to monitor key crop traits, such as canopy cover, LAI, chlorophyll content, plant nutrients, water status, biomass and yield (Horler et al., 1983; Raun et al., 2001; Gutierrez et al., 2010). The vegetation indices built through the combination of spectra in the near-infrared and red channels, such as NDVI, RDVI, and GNDVI, can be used to monitor LAI and canopy cover (Danks et al., 1984; Curran, 1985). The vegetation indices composed of a combination of spectra in the red, blue and infrared channels, such as OSAVI, EVI, RVI, PVI, and DVI, can be used to estimate the chlorophyll content and leaf nitrogen content (Samseemoung et al., 2012; Nigon et al., 2015). The vegetation indices composed of a combination of spectra in the green and red channels, such as the red green ratio index, can be used to determine the carotenoid content. The vegetation indices built based on the difference between two or more spectral channels, such as DVI/EVI, which is sensitive to changes in the soil background, can be used for crop biomass monitoring. The vegetation indices composed of the ratio between two or more spectral channels can indicate the difference between crop growth and crop cover. For example, RVI was used for CCC monitoring. Normalized difference vegetation indices, such as NDVI/RDVI, can reflect crop growth and nutrition. The combination of blue, red and near-infrared channels can eliminate the interference of atmospheric aerosols on vegetation indices, such as OSAVI, which can improve the accuracy of crop growth monitoring and yield prediction. There're advantages of easy computation, low instrumental requirements for the empirical statistical analysis method based on vegetation index, while the limitation include the lack of crop physiological interpretation, and need to be empirically fitted to each particular scenario. In addition to the empirical statistical method for crop phenotyping, the semi-mechanistic model, mechanism model and machine learning are also useful methods. However, the most adopted method for crop phenotyping by UAV remote sensing is mainly empirical statistical model.

**Table 5:** Typical vegetation indices used for field-based phenotyping with UAV platform.

**Vegetation index**	**Formula**	**Related crop traits**	**References**
BGI2 (Blue Green Pigment Index 2)	R_450_/R_550_	LAI, chlorophyll	Aasen et al., 2015
CSI (Canopy Structure Index)	2sSR−sSR^2^ +sWI^2^ WI = R900/R970 SR = R_800_/R_680_	Water	Aasen et al., 2015
DVI (Difference Vegetation Index)	R_nir_−R_red_	Nitrogen, chlorophyll	Jordan, 1969
EVI (Enhanced Vegetation Index)	2.5(R_nir_−R_red_)/(R_nir_+6R_red_−7.5R_blue_+1)	Chlorophyll	Huete et al., 1997
GNDVI (Green Normalized Difference Vegetation Index)	(R_nir_−R_green_)/(R_nir_+R_green_)	LAI, chlorophyll, nitrogen, protein content, water content	Gitelson et al., 1996; Garcia-Ruiz et al., 2013
NDVI (Normalized Difference Vegetation Index)	(R^※^_nir_−R_red_)/(R_nir_+R_red_)	LAI, yield, biomass	Aasen et al., 2015; Zaman-Allah et al., 2015
OSAVI (Optimized Soil-Adjusted Vegetation Index)	1.16(R_800_−R_670_)/(R_800_+R_670_+0.16)	Chlorophyll	Gitelson et al., 1996; Berni et al., 2009b
PRI (Photochemical Reflectance Index)	(R_570_−R_530_)/(R_570_+R_530_)	Chlorophyll, nitrogen, water	Suarez et al., 2009
PSRI (Plant Senescence Reflectance Index)	(R_680_−R_500_)/R_750_	Chlorophyll, nitrogen	Gitelson et al., 1996
PVI (Perpendicular Vegetation Index)	(NIR−aR−b)/$\sqrt{1+a^{2}}$	Chlorophyll	Richardson and Wiegand, 1977
RDVI (Renormalized Difference Vegetation Index)	(R_800_−R_670_)/$\sqrt{{\text{R}}_{800}-{\text{R}}_{670}}$	LAI, biomass, nitrogen	Tucker, 1979
RVI (Ratio Vegetation Index)	R_nir_/R_red_	Water content, yield, chlorophyll, nitrogen	Rondeaux et al., 1996
TCARI (Transformed CAR Index)	3^*^[(R_700_−R_670_)−0.2^*^(R_700_−R_550_)^*^(R_700_/R_670_)]	Chlorophyll	PeÑUelas et al., 1993
VDI(Vegetation Drought Index)	(R_970_−R_900_)/(R_970_−R_900_)	Water stress	Suarez et al., 2009

*R^※^ means spectral reflectance*.

### Crop physiological traits

The reflectance of plant leaves in visible light is affected by the contents of chlorophyll, carotene and lutein in the palisade tissue, whereas the reflectance of plant leaves in the near-infrared band is closely related to the cell structure. A large number of vegetation indices can be built after the empirical treatment of spectral information and can be used to estimate many biophysical properties, such as the chlorophyll, protein content, biomass, malnutrition, crop vigor and water status (Ma et al., 2001; Prasad et al., 2007). For example, the NDVI can reflect the effect of background information on the canopy spectra to estimate crop vigor, biomass, and yield (Hall et al., 2003; Gao et al., 2016). GNDVI and NDWI were used to estimate the leaf chlorophyll content and water status, respectively, (Prasad et al., 2007; Lelong et al., 2008; Gonzalez-Dugo et al., 2014). The accuracy and reliability of the estimation of crop physiological traits were determined by the retrieval model. The most commonly used modeling and analysis method for crop phenotyping by remote sensing is regression analysis, including linear and nonlinear regression. Linear regression methods, including multivariate linear regression (MLR), stepwise linear regression (SLR), and PLSR, are simple and feasible (Capolupo et al., 2015) and have wide applications in quantitative analysis by remote sensing. For example, MLR was used to estimate crop biomass (Rosen et al., 2006; Swain et al., 2010), PLSR was used to estimate the LAI in soybean breeding (Lu et al., 2016), and SLR was used to estimate the leaf chlorophyll content (Berni et al., 2009b). However, the primary objection to linear regression methods is the lack of explanation of crop physiology. In recent years, using nonlinear regression methods, including principal component analysis, artificial neural network, support vector machine and wavelet analysis, data mining has been widely employed to associate hyperspectral information with physiological and biochemical crop parameters (Gong, 1999; Liang, 2005; Wang et al., 2008). However, because there is no explicit regression formula for these nonlinear regression models, it is difficult to obtain a universal analysis model, and the calculation process is more time-consuming, which limits the efficiency and application of these methods. Intercepted photosynthetically active radiation (IPAR) act as an important indicator of photosynthesis and biomass accumulation, methods based optical remote sensing, such as 3D radiative transfer model, Forest Light Interaction Model (FLIGHT) have been adopted to study the fraction of photosynthetically active radiation intercepted (fIPAR) and absorbed (fAPAR). The high-resolution multi-spectral images acquired by UAV provide a rapid and non-destructive way for fiPAR and fAPAR estimation in recent years (Guillen-Climent et al., 2012).

**Figure 8:**
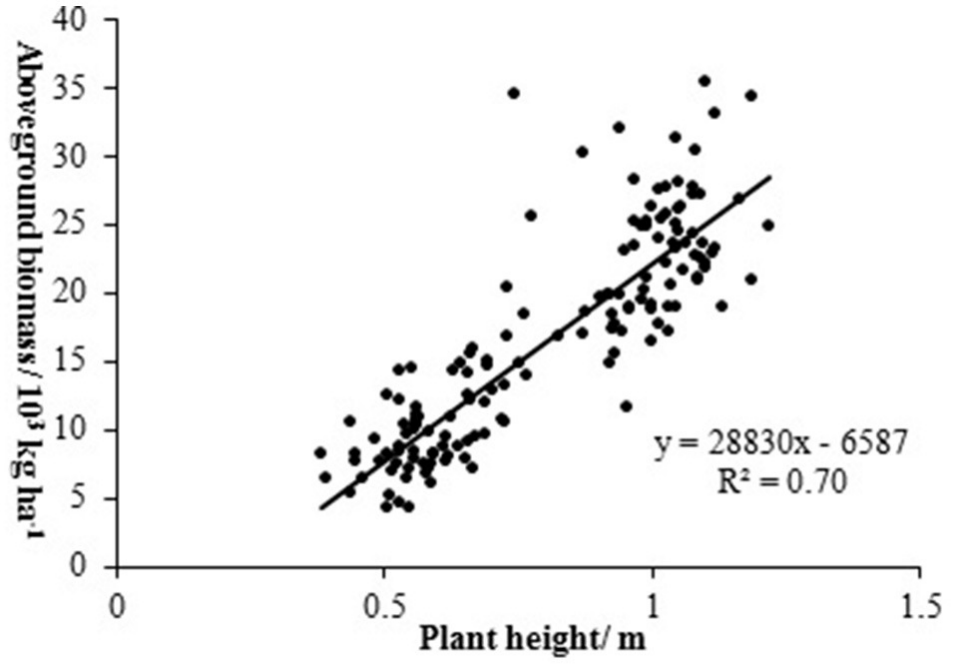
The relationship between plant height and aboveground biomass of soybean (from Lu et al., 2016; permissions for reproduction have been obtained from the copyright holders).

**Figure 9:**
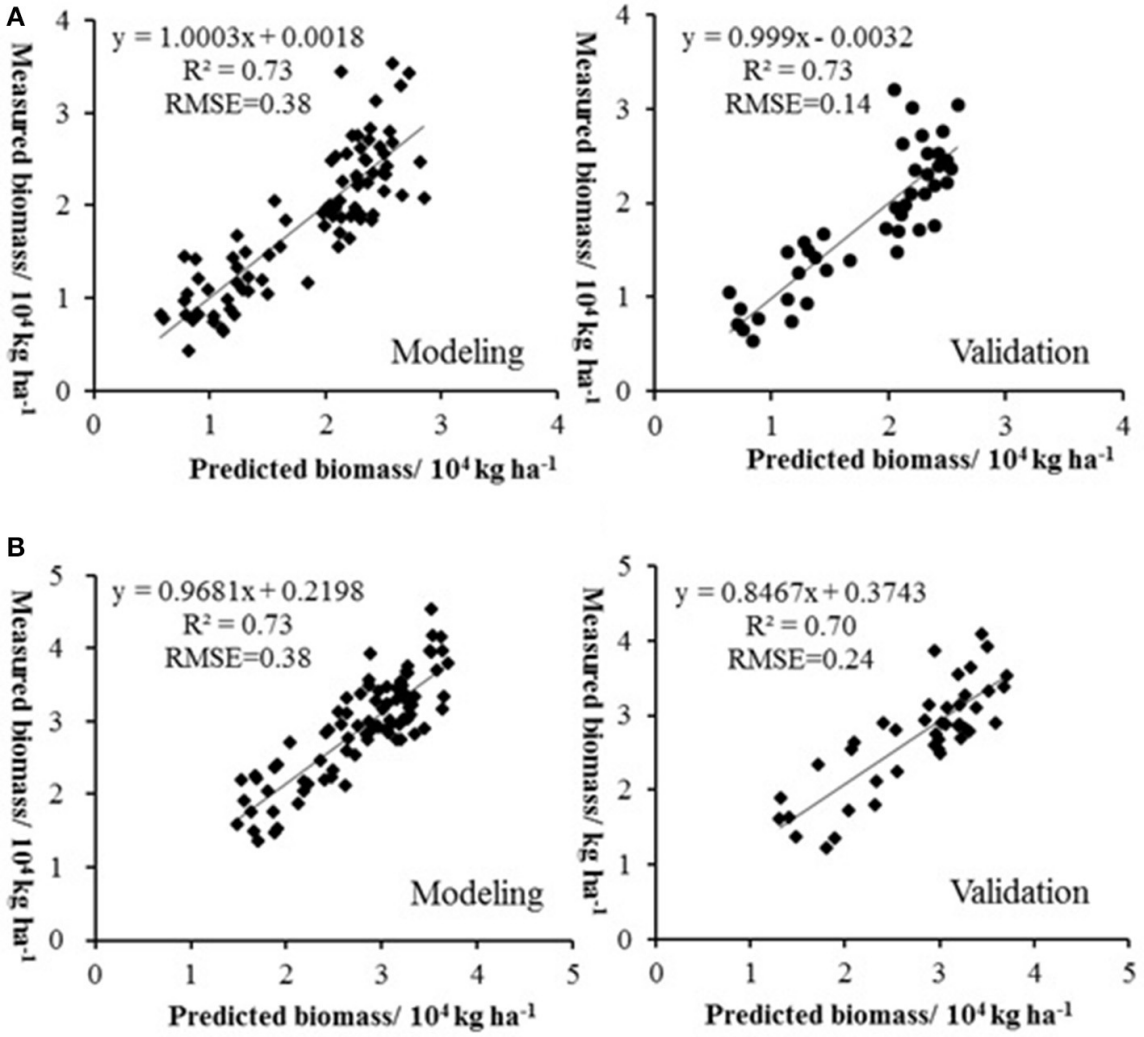
The relationship between predicted and measured biomass in different growth stages. **(A)** The calibration and validation of biomass during the periods of flowering and pod filling; **(B)** The calibration and validation of biomass during the periods of filling and ripening; from Lu et al. (2016); permissions for reproduction have been obtained from the copyright holders.

Biomass is an important indicator of crop growth, which can be used for crop monitoring and yield prediction. The sensors including multispectral camera, hyperspectral imager, LIDAR, combing the methods including empirical statistical analysis of vegetation indices, estimation based on net primary productivity, and crop growth model have been widely deployed for biomass estimation (Hunt et al., 2005; Swain et al., 2010; Wallace et al., 2012; Bendig et al., 2014). However, the method for the estimation of crop biomass by UAV remote sensing is mainly empirical statistical model. In order to improve the accuracy of biomass estimation, the vegetation indices combing plant height have been used for estimating crop biomass. For example, the plant height from crop surface model combing RNDVI were used for barley biomass estimation with the *R*^2^ of 0.84, while the best performance of estimating biomass by single vegetation index got by GRVI and MGRVI with *R*^2^ of 0.60 (Ota et al., 2015). In addition to the retrieval model with single vegetation index, the multiple regression model with several vegetation indices have also been adopted to estimate biomass, which showed good accuracy. For example, in NERCITA's soybean breeding experiment, UAV-based hyperspectral remote sensing was used to estimate soybean biomass in 126 plots by NERCITA and provided a rapid and non-destructive method to estimate crop biomass under complex field environments. As there is a good relationship between soybean plant height and biomass (Figure 8), the plant height combined vegetation indices acquired by a Cubert UHD 185 hyperspectral imager were used to build retrieval models using PLSR to estimate the soybean biomass of a massive germplasm. As the relationship between the canopy spectral characteristics and soybean biomass in different growth stages showed significant differences, the method of “segmentation modeling” was used to estimate soybean biomass under different growth stages. The vegetation indices of OSAVI, R_726_ and NDVI_705_ were used to build a retrieval model to estimate the biomass in soybean breeding during the periods of flowering and pod filling, with the determination coefficient and RMSE of 0.71 and 0.39, respectively. While the RVI, VOG1, NDVI, and Ratio of green peak and red valley (R1) were used to build a retrieval model to estimate the biomass in soybean breeding during the periods of filling and ripening, with a determination coefficient and RMSE of 0.70 and 0.38 (Figure 9; Lu et al., 2016).

### Crop abiotic/biotic stress

Plant physiology research under abiotic or biotic stress conditions usually focuses on the changes of physiological traits and biochemical substances and its agronomy mechanism. Stress factors, including water deficit, low temperature, high temperature, high salinity, environmental pollution, pests and diseases, can have significantly adverse effects on crop growth (Zhao and Qian, 2004; Zarco-Tejada et al., 2012; Nigon et al., 2015). Studying the response of crops to different stress conditions is important for crop cultivation and breeding. As the membrane permeability of plant cells, the chlorophyll content, and peroxidase change under stress conditions, thus, some diseases can be detected by spectroscopy at early growth stage. For example, the UAV-based multi-band imaging sensor was deployed to acquire high-resolution aerial imaging for Huanglongbing (HLB) detection, which yielding that there's significant difference for the 710 nm spectral reflectance and the NIR-R index values between healthy and HLB-infected trees (Garcia-Ruiz et al., 2013). The vegetation indices can be extracted from images acquired by UAV to separate the healthy oil palm and those infested with Phellinus Noxius by visualization, analysis and identification of image processing software, which provide a way for timely detection of pest or disease infections (Samseemoung et al., 2011). The weed can also be separated crops with UAV-deployed multispectral images. For example, the *Silybum marianum* (L.) Gaertn weed was classified using Maximum Likelihood classifier (ML) with multispectral images acquired by a fix-wing UAV. The overall accuracy of classification rates can reach up to 87.04% (Tamouridou et al., 2017). As crop phenotypic information plays an important role in revealing the resistance of crops to stress, rapid phenotyping is essential for agricultural scientists to achieve their goals. Remotely sensed infrared canopy temperatures can provide an efficient method for rapid, non-destructive monitoring of whole-plant response to water stress (Rashid et al., 1999; Gutierrez et al., 2010), which has been widely used to screen drought tolerance varieties. The evapotranspiration can be estimated with thermal infrared images under stress conditions (Grieder et al., 2015). The canopy temperature can also be used for predict crop yield at some occasions. For example, there was a significant positive correlation between lower canopy temperature and higher yield under conditions of high temperature and drought (Reynolds et al., 2007). The stomatal conductance decreased and the leaf temperature increased with stoma closure under osmotic stress caused by excess salinity and high temperature, which can be used to estimate leaf water potential and stomatal conductance (Bowman and Strain, 1988; Wang et al., 2003).

As the crop canopy temperature is related to photosynthesis, the canopy air temperature difference (TD), which is the ratio of the canopy temperature and air temperature, can be used to predict crop yield, for example, there is a significant negative correlation between the TD and yield of hybrid sorghum potato (Chaudhuri and Kanemasu, 1982) and a significant positive correlation between TD and wheat yield under water stress conditions (Rashid et al., 1999; Bellundagi et al., 2013). The water deficit index obtained from thermal imaging data can be used to determine the water status of crop leaves and to estimate the stomatal conductance. The canopy temperature plays an important role in screening drought-resistant varieties by the International Maize and Wheat Improvement Centre (Reynolds et al., 1994). Approximately 60% of the variation of seed yield of different wheat varieties can be explained by high temperature and drought conditions and canopy temperature, which are negatively correlated with the yield of wheat (Olivares-Villegas et al., 2007; Reynolds et al., 2007). Keep (2013) found a difference in canopy temperature between soybean varieties bred during the periods of 1920–2010, which was not affected by the environment. In addition, there is a significant negative correlation between canopy temperature and yield for 2 groups of ripe varieties (Keep, 2013).

The conventional method of measuring the crop canopy temperature in the field is using a handheld thermal imager, which is inefficient. It is difficult to synchronously measure agronomic characteristics in a large area within a short time using a handheld thermal imager, but a UAV equipped with a thermal imaging instrument can rapidly and easily acquire thermal imaging data for crop growth monitoring in large areas to indirectly monitor crop growth status. The use of UAVs equipped with thermal imagers to monitor the crop canopy temperature, stomatal conductance and leaf water status of wheat, maize and sorghum showed high accuracy compared with the data observed in the field, while such indicators of cotton acquired by UAV-based thermal imagers showed lower accuracy (Berni et al., 2009b; Zarco-Tejada et al., 2012; Colomina and Molina, 2014).

### Crop potential yield prediction and nutrient monitoring

Crop yield, which is closely related to the development and differentiation of organs and the distribution and accumulation of photosynthetic products, is the core focus of crop science research. The traditional methods for crop yield estimation are the use of manual surveys or establishing the relationship between agronomic factors or climatic factors and crop yield using statistical analysis methods (Overgaard et al., 2010; Swain et al., 2010). Many observations and samplings in field experiments are required to determine the parameters of the yield prediction model, which is laborious and time-consuming. As some factors are difficult to quantify, it is difficult to promote the use of these models due to the poor adaptability to different areas. Yield prediction by remote sensing is defined as building the relationship between the canopy spectra and crop yield based on the biological characteristics of crops for yield prediction using spectral data at different crop growth stages (Swain et al., 2010). Improving the accuracy and adaptability of the yield estimation model is a prerequisite for the application of UAV remote sensing. Crop yield can be predicted by combining the plant physiological parameters with the vegetation indices. The commonly adopted plant physiological parameters and remote sensing parameters for building yield prediction models include the length of the growth period, chlorophyll content, LAI, aboveground biomass, spectral reflectance and vegetation indices (Filella et al., 1995). Crop yield can be predicted by constructing a remote sensing inversion model containing a variety of vegetation indices; however, the accuracy of crop prediction using the structural index is low due to the effects of terminal water stress (Chaudhuri and Kanemasu, 1982). The canopy temperature, which is related to yield to a certain extent, and carotenoids pigment indices, such as the PRI and chlorophyll absorption reflectance index (CARI), are suited to screening complex traits, such as crop yield (Gonzalez-Dugo et al., 2015). For example, the wheat yield was predicted based on canopy temperature as low temperature increases the yield under adequate water supply and water limitation conditions (Olivares-Villegas et al., 2007). The chlorophyll content can also be used to predict crop yield because there is a relationship between the chlorophyll content and photosynthesis that is related to yield. The accuracy of yield prediction models improves with increasing number of modeling parameters; however, the yield prediction models using spectral reflectance characteristics and canopy temperature usually focus on 2~3 bands and lack adaptability. Therefore, it is important to build crop yield prediction models that combine crop physiology and remote sensing parameters to improve the accuracy of yield prediction by UAV-RSP.

In recent years, the spectral characteristics of components, such as plant chlorophyll, nitrogen and water, were identified, which enabled the estimation of the biochemical contents of crops by remote sensing and estimation of crop nutrition under different environmental conditions (Swain et al., 2007; Wang et al., 2008). Leaf nitrogen concentration (LNC) is an important indicator of nitrogen (N) status for assessing dynamic regulation and predicting crop yield. Research progress of remote sensing technology for LNC estimation have been achieved in the past years. The adopted algorithms and vegetation indices for building remote sensing analytical models significantly affect the accuracy of field phenotyping using UAV-RSPs. As models for phenotypic information analysis of massive cultivars in breeding plots using unmanned aerial vehicle remote sensing are sensitive to climate and soil conditions, developing strategies for LNC estimation for different crops under different environmental conditions is helpful for crop growth monitoring. In NERCITA's study, four chemometric techniques, including stepwise multiple linear regression (SMLR), partial least squares regression (PLSR), back propagation neural network (BPN), and support vector machines regression (SVR), were adopted with 13 key spectral features to build LNC models. The results indicated that partial least squares regression (PLSR) and support vector machines regression (SVR) performed better than the other two methods, with *R*^2^ values in the calibration set of 0.82 and 0.81 and the normalized root mean square error (NRMSE) values in the validation set of 5.48 and 5.94%, respectively (Li Z. et al., 2016).

## Discussions

### UAV-RSPs and deployed sensors

UAV remote sensing platforms have become an effective way to rapidly acquire ground information, especially in the field of crop growth monitoring. It is necessary to adjust the flight altitude and flight speed based on the actual conditions due to the complexity of the farmland environment. In contrast, the flight speed and altitude must be lower to obtain detailed information about fruit growth in the bush (Mathews and Jensen, 2013). Frequently used UAV-RSPs, especially multi-rotor UAVs, such as the quadcopter UAV model md4-1000, are unmanned gyroplanes. Gyroplanes have the advantages of adjustable flight speed and height, the ability to take-off and land vertically, freedom from site condition restrictions, and suitability for the observation of precise farmland information (Hardin and Jensen, 2011). The flight altitude of multi-rotor UAVs ranges from 20 to 100 m, guaranteeing that the spatial data resolution of the optical sensors can reach the centimeter level for accurate identification of crop individuals. The flight altitude ranges from 300 to 1,000 m for most fixed-wing UAVs, and the biggest problem for the fixed-wing UAVs is that a minimum flight speed before they stall is required. The operation of fixed-wing UAVs is more complicated than that of multi-rotor UAVs, which make it more dangerous. The disadvantages limit the wide application of fixed-wing UAVs for data acquisition in field phenotyping. Most UAVs are equipped with automatic driving systems. The automatic adjustment of flight height, position and attitude is conducted using GPS/INS and the pressure gauge mounted on the UAV, which can reduce the intensity of manual control and avoid the impact of human factors on flight safety (Pajares, 2015). The payload can reach 3–5 kg for the most multi-rotor and fixed-wing UAVs. Additionally, the UAV body must be larger when the payload exceeds 5 kg; it is necessary for the UAV to be equipped with an ejection frame at the specialized site for take-off and to conduct artificial judgment to open the parachute, which increases the complexity and difficulty of operating fixed-wing UAVs. In general, multi-rotor UAVs are more stable and convenient and are more suitable as a platform for field-based crop phenotyping. Nevertheless, the limited extension that rotary wings can cover by speed and autonomy is the problem for its application. In the future, multi-rotor UAVs will be able to provide more than 1 h of continuous flight as battery technology matures.

UAVs are currently equipped with optical sensors with wavebands ranging from visible light to near-infrared, such as multispectral sensors, conventional digital cameras, and hyperspectral sensors. The problems with sensors deployed by UAV-RSP are as follows: (1) the lack of sensors for field-based crop phenotyping. The existing optical sensors are difficult to use to obtain quantitative crop information and are usually employed for qualitative analysis. For example, an 8-bit storage format is used for imaging by the Tetracam ADC-lite camera, which can result in a very small change in spectral difference between crops and affect the accuracy of the subsequent quantitative analysis. The commonly used digital cameras lack camera calibration and radiometric calibration, which can affect the accuracy of the geometric parameter analysis. Unmanned airborne hyperspectral imaging, including linear push broom imaging and staring imaging, has gradually gained the attention of experts and scholars. The linear push broom imaging hyper-spectrometer is more mature, while the staring hyperspectral imager was developed for unstable platforms in the last 2 years. The biggest problem with the linear array push broom spectrometer is that it is difficult to guarantee accurate geometric calibration. The spatial resolution of the spectrometer can reach the centimeter level, but the vibration of the UAV platform and dynamic jitter can cause obvious geometric distortion in the absence of high-precision positioning and orientation system (POS) or inertial navigation system (INS). The vibration can be reduced by a shock absorbers, or an active stabilization platform. However, it is difficult for the geometric calibration accuracy to reach the centimeter level, which can seriously affect the application of this type of sensor. Therefore, it is necessary to develop sensors to be deployed by micro-UAV for field-based crop phenotyping. (2) The current sensor acquisition control system and UAV flight control system are not integrated. Currently, the UAV and the deployed sensors are from different manufacturers. Therefore, the flight control system of the UAV does not provide an interface for the sensors, and the self-control system, acquisition and storage unit are equipped by the sensors without the interface and without a UAV flight control system. It is necessary to dynamically control the sensors using the UAV flight control system in the process of UAV remote sensing data acquisition to meet the needs of data acquisition and to avoid redundant data acquisition by the sensors.

### Airspace regulations for the application of UAVs

The UAVs have the advantages of flexible, real-time and non-destructive for crop phenotyping, however, UAVs must follow strict management rules to ensure their security as well as the sustainable development of the UAV aviation industry. As the flight plan needs to be permitted by aviation regulators, which usually take more than 3 days in some countries. For example, permissions from more than 3 regulatory agencies, such as air force, civil aviation, police, are needed to conduct UAV flight in China, which is time consuming. The flight plan including the flight time, flight altitude UAV driver, flying area and purpose of flight etc. must be submitted. As the plan for the acquisition of UAV remote sensing information was usually scheduled based on the weather and crop growth stages, it's hard to propose advanced declaration for conducting the research of field phenotyping. In addition, the frequent flight may be needed in the research, the repeated declarations are trouble. Here we present a summary of the main regulation for UAVs in different countries. As one of the most developed countries in the aviation industry, the regulations of UAVs in the United States are in a relatively advanced position. UAVs were admitted to the National Aeronautics and Space Administration (NASA) in the United States in 1990, and UAV regulation found a balance between caution and openness by the Federal Aviation Administration (FAA) with the increase in the civilian UAV operating frequency. There are three types of UAVs that need to be supervised: public aircraft, civil aircraft, and model planes. The US FAA announced an online registration of commercial drones and small UAVs in 2016, with equal registration costs for recreational drones. The web version of the registration system was designed to reduce the time required for a large number of UAV operators to register commercial UAVs. Recently, the US FAA proposed a regulatory requirement that a UAV must be in sight of its operator, which limits the wide application of UAVs for large area crop surveys, long distance inspection of pipelines and delivery. To address this limitation, an air traffic management system was developed with the cooperation of NASA, companies, universities and government agencies to allow the operator to use a tracking system so that the UAV can fly out of sight of the operator while avoiding sensitive areas, such as airports and crowded areas. The European Aviation Safety Agency (EASA) issued a notice of advanced system revision (A-NPA2015-10) named “the framework of the UAV operation rules and regulations” on July 30, 2015. The A-NPA provided a detailed operational regulatory framework with low risk proposed by EASA. The supervision by EASA was based on the performance and risk of UAVs, which can be divided into three categories: (1) open class (low risk); (2) franchise operation (medium risk); (3) validation class (high risk). The regulatory framework of EASA is based on the operational risk of the UAV. In addition to the regulations based on risk management, there are regulations based on certification for UAVs, which are similar to those for manned spacecraft (Stöcker et al., 2017). The Civil Aviation Administration of China (CAAC) stipulates that institutions and individuals using UAVs must apply for specialized airspace based on the “General Aviation Flight Control Ordinance” and must obey flight activity management rules to ensure flight safety. The management level of production and the possession and use of UAVs greatly affects the market size of UAV applications, which include UAV remote sensing and field-based crop phenotyping.

### Methods and accuracy of field-based crop phenotyping

Methods for crop phenotype analysis from remote sensing include direct monitoring, image classification, concurrent comparison, empirical statistics modeling, physical reversion modeling, machine learning, and time series analysis of remote sensing parameters (Singh et al., 2016; Virlet et al., 2017). However, the commonly adopted methods for crop phenotyping by UAV remote sensing technology are empirical statistical analysis at present. The other equally important methods for the analysis of remote sensing data haven't been widely used in the research, only few articles present these methods for crop phenotyping by UAV. The statements based on literature survey and case study by NERCITA indicated that the high accuracy for estimating field-based crop phenotypic traits can be attained, however, the high accuracy just can be attained in special occasions. The research that analyze LAI, canopy chlorophyll content and leaf nitrogen content using remote sensing data has been fully developed. The determination coefficient of retrieval models can exceed 0.85 (Rosen et al., 2006; Berni et al., 2009b; Ballesteros et al., 2014; Bendig et al., 2014), indicating that the estimated phenotypic information is in good agreement with the measured values. The reason for the high accuracy is that the spectral characteristics of the leaves are related to the abovementioned indicators and can be used to directly reflect the growth information. Unfortunately, it's hard to propose the universal models or general methods for retrieving crop phenotypic traits with good accuracy by remote sensing among different types of crops so far.

As the vegetation index is an important cause of the differences in remote sensing for retrieving crop phenotypic traits, the combination of spectral bands can affect the retrieval accuracy. The retrieval accuracy of complex traits based on remote sensing in the surveyed literatures remains low. The determination coefficient of retrieval models for predicting crop yield and biomass is usually less than 0.70 (Overgaard et al., 2010) due to the indirect relationship between remote sensing information and complex crop traits. Complex crop traits are influenced by the genome and environment, while the spectral reflectance and vegetation indices (e.g., NDVI, RVI) are indirectly related to these traits. Crop traits such as LAI and fraction of absorbed photosynthetically active radiation are more related to crop yield; however, it is difficult to use crop traits directly related to spectral characteristics to predict crop yield due to the heterogeneity of the earth's surface, the influence of crop type and environment, and the uncertainty of remote sensing extraction.

The basic research ideas for field-based crop phenotyping by UAV-RSP are similar, including retrieving key crop traits, such as crop structure, biochemical content, biomass, yield, lodging, diseases and pests, from remote sensing data (Sankaran et al., 2015b). Most retrieved crop traits are at the canopy scale and can indicate crop growth; however, there is a need to acquire physiological and ecological characteristics of individual plants and even organs that cannot be resolved by UAVs. Therefore, new sensors and physical reversion models are needed to synchronously monitor the key phenotypic information of crops at the micro and macro scales.

### Challenge for “big” data processing

Compared with the rapid development of sensor and hardware platforms, the efficiency and function of image processing are insufficient, especially the rapid processing ability of software in the field. In addition, large amounts of data acquired by hyperspectral imagers and LIDAR need to be processed (Zhao and Qian, 2004). As image processing for hyperspectral and LIDAR data is complex, there is an urgent need to develop software with the ability to perform high-speed and accurate image processing to improve the efficiency and accuracy of data processing. The data processing flow is based on aerial remote sensing image processing for most existing UAV remote sensing processing software, without consideration of the specialized characteristics of UAV remote sensing. The aspects reflecting the difference between aerial remote sensing and UAV remote sensing include few ground control points, a lack of strict internal calibration parameters in the deployed sensors, and a lack of a high-precision POS system for UAV remote sensing, which prevents the existing aerial remote sensing processing system from being directly applied to UAV remote sensing data processing. Digital image processing software, such as Agisoft PhotoScan Professional Edition (Agisoft LLC, St. Petersburg, Russia) and Pixel4D (Lausanne Switzerland), which can be used for geometric correction and mosaicking, has rapidly developed in recent years; however, hyperspectral images and LIDAR data cannot be processed using commercial processing software due to the lack of a special module for remote sensing information analysis. The efficiency of data processing software must be improved to satisfy the requirements of rapid and accurate data processing and analysis.

The geometric correction and radiometric calibration constitute the most demanding work for UAV remote sensing data processing, which is time-consuming. A mature remote sensing photogrammetry method was adopted for geometric correction, which requires many points matching the same name, point cloud generation and correction of ortho images. In addition, it is difficult to batch process because the spectral and spatial resolution of each sensor is different, which reduces the processing efficiency. It is necessary to develop UAV remote sensing output processing methods based on cloud computing or high-performance computing to process remote sensing data online and to avoid the low efficiency caused by data processing using stand-alone software. Additionally, there is a need to develop specialized analytical tools for sensors deployed in crop phenotyping research using UAV-RSPs for wide applications by crop breeders. The application of UAV-RSPs for field-based crop phenotyping can promote the association analysis of genomes and phenotypes to improve crop breeding efficiency.

## Conclusion

Field-based crop phenotyping by UAV-RSPs has become a hot research topic in recent years. Approximately 88.5% of the surveyed literature focused on field phenotyping using UAV-RSPs was published in the last 5 years. The current status and perspectives of UAV-RSPs for FBP are follows:

1. UAV-RSP can be as a powerful tool for field-based phenotyping with the advantages of high operation efficiency, low cost, suitability for complex field environments, and high resolution. The multi-rotor UAV is the mostly adopted UAV-based phenotyping platform in the recent years. There's potential for UAV-RSP acting as an alternative to the traditional methods for crop growth monitoring, yield prediction and variety selection. The digital camera, multispectral camera, hyperspectral camera, thermal infrared imager and LiDAR have been widely used to field-based phenotyping. The adoption of multi-sensors coupled with advanced data analysis methods for retrieving crop phenotypic traits are the research hotspots in recent years.
2. The crop phenotype that can be acquired by UAV-RSP include, but are not limited to, geometric traits, canopy spectral texture, physiological traits, abiotic/biotic stress response, plant nutrition, and yield. Unfortunately, there is still a lack of validation for field-based phenotyping by UAV with a large group of crop varieties. There exists difference for the accuracy of field-based phenotyping using UAV remote sensing among phenotypic traits, which was caused by the variation of sensor type, climate, crop growth stages and crop type. Research focused on crop phenotypic traits that are directly related to the canopy spectral information has been conducted and has shown good accuracy under certain conditions, while there is low accuracy in the research on the non-destructive acquisition of complex traits that are indirectly related to the canopy spectral information.
3. The limiting factors for UAV-based field phenotyping include the low capability of UAVs, the strict airspace regulations, the lack of methods for fast data processing and models for estimating complex traits under different environmental conditions. Improving the performance of UAVs, reducing the cost of sensors, speeding up data processing and developing strategies for analyzing crop phenotype by remote sensing are future trends. Fortunately, it is expected that with the advancement of UAVs with larger payload and longer endurance, low-cost sensors, improved image processing methods for “Big” data, and effective airspace regulations, there's potential for wider applications about the UAV-based field phenotyping.

## Author contributions

GY, JL and CZ conducted the literature survey and drafted the article; HYu, BX, XY, DZ, XZhang, RZ, HF and XZhao provided the data and figures about the field-based phenotyping by NERCITA; Zhenhong Li, HY, Zhenhai Li, HL and HYang gave valuable comments to the manuscript and carried out critical revisions. All authors gave final approval for publication.

### Conflict of interest statement

The authors declare that the research was conducted in the absence of any commercial or financial relationships that could be construed as a potential conflict of interest.

## Abbreviations

AIC: Akaike information criterion
BGI2: Blue green pigment index2
BPN: Back propagation neural network
CAAC: Civil aviation administration of China
CARI: Chlorophyll absorption reflectance index
CCC: Crop canopy cover
CHM: Crop height model
CSI: Canopy structure index
DEM: Digital elevation model
DSM: Digital surface model
DVI: Difference vegetation index
EASA: European aviation safety agency
EVI: Enhanced vegetation index
FAA: Federal aviation administration
FBP: Field-based phenotyping
FBPPS: Field-based phenotyping platforms
GNDVI: Green normalized difference vegetation index
HTPPs: High-throughput phenotyping platforms
INS: Inertial navigation system
LAI: Leaf area index
LIDAR: Laser intensity direction and ranging
LNC: Leaf nitrogen concentration
MLR: Multivariate linear regression
NASA: National aeronautics and space administration
NDVI: Normalized difference vegetation index
NERCITA: National engineering research center for information technology in agriculture
NLI: Nonlinear index
OSAVI: Optimized soil-adjusted vegetation index
PLSR: Partial least squares regression
POS: Positioning and orientation system
PRI: Photochemical reflectance index
PSRI: Plant senescence reflectance index
PVI: Perpendicular vegetation index
RDVI: Renormalized difference vegetation index
RVI: Ratio vegetation index
SAR: Synthetic aperture radar
SAVI: Soil-Adjusted Vegetation Index
SLR: Stepwise linear regression
SMLR: Stepwise multiple linear regression
SR: Simple ratio
SVR: Support vector machines regression
TCARI: Transformed CAR Index
TD: Temperature difference
TVI: Triangular vegetation index
UAV: Unmanned aerial vehicle
UAV-RSP: Unmanned aerial vehicle remote sensing platforms
VDI: Vegetation drought index.
